# A novel interaction between dengue virus nonstructural protein 1 and the NS4A-2K-4B precursor is required for viral RNA replication but not for formation of the membranous replication organelle

**DOI:** 10.1371/journal.ppat.1007736

**Published:** 2019-05-09

**Authors:** Anna Płaszczyca, Pietro Scaturro, Christopher John Neufeldt, Mirko Cortese, Berati Cerikan, Salvatore Ferla, Andrea Brancale, Andreas Pichlmair, Ralf Bartenschlager

**Affiliations:** 1 Department of Infectious Diseases, Molecular Virology, University of Heidelberg, Heidelberg, Germany; 2 Max-Planck Institute of Biochemistry, Innate Immunity Laboratory, Martinsried, Germany; 3 School of Medicine, Institute of Virology, Technical University of Munich, Munich, Germany; 4 School of Pharmacy & Pharmaceutical Sciences, Cardiff University, Cardiff, United Kingdom; 5 German Center for Infection Research (DZIF), Munich Partner Site, Munich, Germany; 6 German Center for Infection Research (DZIF), Heidelberg Partner Site, Heidelberg, Germany; The University of Chicago, UNITED STATES

## Abstract

Dengue virus (DENV) has emerged as major human pathogen. Despite the serious socio-economic impact of DENV-associated diseases, antiviral therapy is missing. DENV replicates in the cytoplasm of infected cells and induces a membranous replication organelle, formed by invaginations of the endoplasmic reticulum membrane and designated vesicle packets (VPs). Nonstructural protein 1 (NS1) of DENV is a multifunctional protein. It is secreted from cells to counteract antiviral immune responses, but also critically contributes to the severe clinical manifestations of dengue. In addition, NS1 is indispensable for viral RNA replication, but the underlying molecular mechanism remains elusive. In this study, we employed a combination of genetic, biochemical and imaging approaches to dissect the determinants in NS1 contributing to its various functions in the viral replication cycle. Several important observations were made. First, we identified a cluster of amino acid residues in the exposed region of the *β-ladder* domain of NS1 that are essential for NS1 secretion. Second, we revealed a novel interaction of NS1 with the NS4A-2K-4B cleavage intermediate, but not with mature NS4A or NS4B. This interaction is required for RNA replication, with two residues within the connector region of the NS1 “*Wing*” domain being crucial for binding of the NS4A-2K-4B precursor. By using a polyprotein expression system allowing the formation of VPs in the absence of viral RNA replication, we show that the NS1 –NS4A-2K-4B interaction is not required for VP formation, arguing that the association between these two proteins plays a more direct role in the RNA amplification process. Third, through analysis of polyproteins containing deletions in NS1, and employing a *trans*-complementation assay, we show that both *cis* and *trans* acting elements within NS1 contribute to VP formation, with the capability of NS1 mutants to form VPs correlating with their capability to support RNA replication. In conclusion, these results reveal a direct role of NS1 in VP formation that is independent from RNA replication, and argue for a critical function of a previously unrecognized NS4A-2K-NS4B precursor specifically interacting with NS1 and promoting viral RNA replication.

## Author summary

Dengue virus (DENV) is one of the most prevalent mosquito-transmitted human pathogens. The only licensed vaccine has limited efficacy and an antiviral therapy is not available. The multifunctional non-structural protein 1 (NS1) of DENV is secreted from infected cells, counteracts antiviral immune response and contributes to the pathogenesis of DENV infection. In addition, NS1 is essential for the viral replication cycle but the underlying mechanism is unknown. Here we determined the viral interactome of NS1 and identified a novel interaction of NS1 with the NS4A-2K-4B cleavage intermediate, but not with NS4A and NS4B. This interaction is required for RNA replication. Additionally, we identified a domain in NS1 important for efficient secretion of this protein. Finally, we demonstrate that NS1 is required for the biogenesis of the membranous DENV replication organelle. This function does not require RNA replication and is independent from NS1 interaction with NS4A-2K-4B. Our results provide new insights into the role of NS1 in DENV RNA replication and establish a genetic map of residues in NS1 required for the diverse functions of this protein. These results should aid in the design of antiviral strategies targeting NS1, with the aim to suppress viral replication as well as severe disease manifestations.

## Introduction

Dengue virus (DENV), the causative agent of dengue fever, is the most prevalent arbovirus infecting humans worldwide. It is estimated that all four serotypes of DENV combined are responsible for ~390 million infections annually, leading to ~20,000 deaths [1]. Despite many efforts, no antiviral therapy against DENV is available to date and the only approved vaccine has limited efficacy and depends on baseline serostatus of the vaccine recipient [2]. DENV belongs to the *Flavivirus* genus in the *Flaviviridae* family and is a small enveloped virus with a single-stranded RNA genome of positive polarity and a length of ~10,700 nucleotides. Upon binding to various attachment factors on the cell surface, DENV enters the cell mainly via clathrin-mediated endocytosis, although other entry routes have been described [3]. Upon fusion with the endosomal membrane, the viral RNA is released into the cytoplasm and translated in a cap-dependent manner. The translation product is a polyprotein that is cleaved co- and post-translationally by viral and cellular proteases into 10 proteins. These are the three structural proteins capsid (C), envelope (E) and premembrane (prM) and the seven nonstructural proteins NS1, NS2A, NS2B, NS3, NS4A, NS4B and NS5. The nonstructural proteins induce massive remodeling of ER membranes, manifesting as convoluted membranes and vesicle packets (VPs). While the function of convoluted membranes is still not clear, VPs most likely are the site of viral RNA replication. Consistently, VPs are clustered ER membrane invaginations with each vesicle connected to the cytoplasm via a ~11 nm pore [4,5]. Several enzymatic functions have been identified amongst the nonstructural proteins. These comprise an RNA-dependent RNA polymerase and a methyltransferase activity for NS5, a serine protease in the amino-terminal region of NS3, which requires NS2B as a protease cofactor, and helicase, NTPase and RNA triphosphatase activities in the carboxy-terminal region of NS3. The functions of the small transmembrane proteins NS2A, NS4A and NS4B, which are all essential for virus replication, are much less understood, although they have been proposed to participate in the modification of intracellular membranes and organelles [6–9] and in counteracting host immune response [10–15].

DENV NS1 exerts an amazing array of different functions. On one hand it is required for RNA replication and assembly/release of virus particles [16–19]; on the other hand NS1 is secreted from infected cells as lipid-containing hexamer [20]. This extracellular form of NS1 plays a critical role in immune evasion of the complement system and contributes to dengue pathogenesis, most likely by triggering the release of vasoactive cytokines from immune cells. These cytokines are thought to induce vascular leakage, which is a hallmark of severe dengue [21].

**Fig 1:**
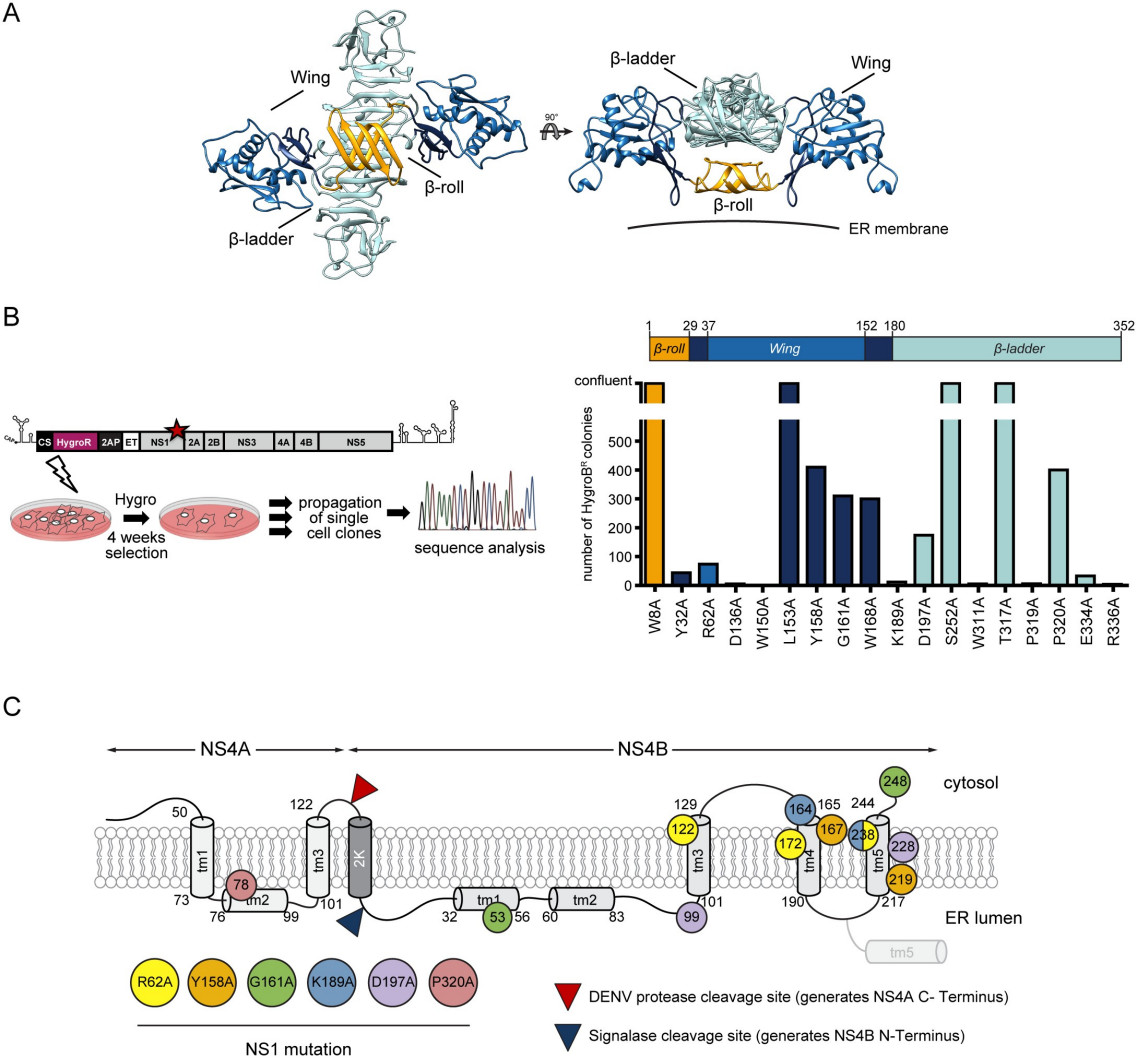
A forward genetic screen identifies pseudoreversions in NS4A and NS4B compensating replication-inactivating mutations in NS1. (A) Homology model of the NS1 dimer based on the DENV NS1 structure (PDB 4O6B) with missing residues modelled according to the ZIKV NS1 structure (PDB 5K6K). The model was built using the MOE 2015 software package and molecular graphics were prepared with UCSF Chimera [66]. *Wing*, *β*-*ladder* and *β*-*roll* domains are shown in blue, turquoise and orange, respectively. The connector subdomains in *Wing* domain are shown in dark blue. The membrane is indicated with a horizontal line. (B) Left panel: schematic of the experimental approach used to select for pseudoreversions rescuing replication of NS1 mutants. Point mutations in NS1 were inserted into a selectable subgenomic DENV replicon (sgDVH2A) encoding a hygromycin phosphotransferase gene (HygroR) downstream of by the cyclisation sequence residing in the capsid-coding region (CS) and upstream of the 2A protease of *Thosea asigna* virus (2AP) and the signal sequence of NS1 corresponding to the last 24 amino acid residues of E (ET). VeroE6 cells were electroporated with selectable replicon RNAs containing given mutations and cultured in the presence of hygromycin B. After three to four weeks, single cell clones were propagated; total RNA was extracted and amplified for sequence analysis. Right panel: number of hygromycin B-resistant cell colonies obtained after selection for each primary mutation in NS1 (indicated at the bottom) with the color of the bar corresponding to the color of the NS1 domain into which the respective mutation was introduced. (C) Schematic summarizing the localization of second site mutations in the NS4A-2K-NS4B polyprotein that is drawn according to its hypothetical membrane topology [7,36]. The circle color of primary mutations in NS1 (lower left) corresponds to pseudoreversions identified for each respective mutant.

NS1 is inserted into the ER lumen via a 24 amino acid residues long signal sequence corresponding to the carboxy-terminus of E. Upon removal of the signal sequence by the host cell signalase and cleavage by an unknown protease at the NS1-NS2A junction, NS1 rapidly dimerizes [22]. The structure of the NS1 dimer is composed of three distinct domains (Fig 1A): first, a *“β-roll”* domain (amino acids 1 to 29) composed of two β-hairpins; second, the “W*ing*” domain (amino acids 30 to 180) formed by the α/β subdomain (amino acids 38 to 151) and the connector subdomains (amino acids 30 to 37 and 152 to 180); and third, the central *“β-ladder”* domain (amino acids 181 to 352) [23]. The connector subdomain and the *β-roll* domain create a hydrophobic surface likely allowing NS1 interaction with the ER membrane [23].

Although many studies confirmed that NS1 is indispensable for viral RNA replication, the underlying mechanism remains obscure. NS1 colocalizes with dsRNA in infected cells and clusters closely to VPs, presumably at the ER luminal surface of the VPs [24], or even inside the vesicles [24,25]. Given this localization and the ability of NS1 to remodel liposomes, NS1 was proposed to participate in the formation or stabilization of membranous viral replication organelles, possibly by interaction with NS4B and/or NS4A [19,23,26,27]. In addition, multiple cellular proteins interacting with NS1 have been identified, including ribosomal proteins, subunits of the oligosaccharyltransferase and the chaperonin TRiC/CCT complex [28,29], suggesting that functions executed by NS1 are mediated, at least in part, by recruited cellular proteins.

With the aim to decipher the mechanism by which NS1 supports the DENV replication cycle we have previously performed a genetic screen and identified a set of mutants that are either impaired in the production of infectious virus particles or RNA replication [17]. While in this previous study we unraveled how NS1 contributes to virus particle assembly and release, in the present study we investigated replication-impaired NS1 mutants with respect to interaction with viral proteins, NS1 secretion and the formation of VPs. We identified a NS1 –NS4B genetic complementation group and determined the viral NS1 interactome. Moreover, we identified a novel interaction between NS1 and the NS4A-2K-4B cleavage intermediate and characterized the role of this interaction for viral RNA replication, NS1 secretion and VP formation. Overall, our results provide a comprehensive map of NS1 determinants required for the multi-functionality of this protein in the DENV replication cycle and demonstrate the indispensable role of NS1 in the formation of the viral membranous replication organelle.

## Results

### A forward genetic screen identifies a NS1 –NS4B complementation group

With the aim to establish a genetic complementation map of DENV NS1 that might inform about its viral interaction partners, we took advantage of 18 alanine substitutions in NS1 that severely impair virus replication [17]. We selected for second-site compensatory mutations by using a selectable subgenomic DENV replicon encoding the hygromycin phosphotransferase gene (sgDVH2A) (Fig 1B, left panel). Single amino-acid substitutions in NS1 that inhibit RNA replication were inserted into this replicon and *in vitro* transcribed RNAs were transfected into VeroE6 cells cultured in the presence of hygromycin B. After three to four weeks, growth of well-isolated single cell clones became apparent for mutants Y32A, R62A, D136A, W150A, Y158A, G161A, W168A, K189A, D197A, W311A, P319A, P320A, E334A and R336A, however in the case of W150A, W311A, E334A and R336A no viable cell clones were successfully propagated. For mutants W8A, L153A, S252A and T317A a high number of colonies was obtained and therefore those mutants were excluded from subsequent analyses (Fig 1B, right panel).

To identify second-site mutations, total RNA was extracted from single cell clones and cDNA fragments spanning the complete non-structural genome region were amplified by RT-PCR. The PCR products were sequenced, examining at least two independent cell clones for each mutant (except Y32A, where only one viable cell clone could be isolated). Besides the D136A and P319A mutations that consistently reverted to wildtype, and D197A that reverted in one instance, the original NS1 alanine substitution was retained and a second-site mutation was identified on the same amplicon. The only exception was the W168A mutation for which no additional mutations were found in the sequenced region (Table 1). Interestingly, the majority of second-site mutations mapped to the NS4B coding region (Fig 1C), an observation that is consistent with the proposed role of the NS1—NS4B interaction in the DENV replication cycle [26]. In addition, we detected second-site mutations in NS4A, NS2A and NS5 (Table 1).

**Table 1:** Summary of second-site mutations and their correspondence to primary NS1 mutations.

Original NS1 mutation	Original Codon	Mutated Codon	Selected mutation			
			protein	aa change	nt change	frequency
**Y32A**	TAC	GCC	**NS2A**	T115S	ACC->TCC	1/1
**R62A**	CGT	GCC	**NS4B**	S238F	TCT->TTT	1/3
			**NS4B**	G122R	GGG->AGG	1/3
			**NS4B**	M172L	ATG->TTG	1/3
**D136A**	GAT	GCC	**NS1**	A136D^†^	GCC->GAC	4/4
**Y158A**	TAT	GCC	**NS4B**	E167H	CAG->CAT	1/3
			**NS1**	M275L	ATG->TTG	1/3
			**NS4B**	V219A	GTG->GCG	1/3
**G161A**	GGA	GCC	**NS1**	D180E	GAC->GAA	1/3
			**NS4B**	R53K	AGA->AAA	1/3
			**NS4B**	R248K	AGG->AAG	1/3
**K189A**	AAA	GCA	**NS4B**	S238F	TCT->TTT	1/2
			**NS4B**	F164L	TTT->CTT	1/2
**D197A**	GAT	GCC	**NS4B**	S228C	AGT->TGT	1/3
			**NS4B**	Y99H	TAC->CAC	1/3
			**NS1**	A197D^†^	GCC->TCC	1/3
**P319A**	CCA	GCC	**NS1**	A319P^†^	GCC->CCC	3/3
**P320A**	CCG	GCC	**NS4A**	I78L	ATA->TTA	1/2^‡^
			**NS5**	A19S	GCA->TCA	1/2^‡^

For each NS1 mutant, one to four cell clones were isolated and derived cDNA was subjected to nucleotide sequence analysis. Frequency indicates in how many of the analyzed clones the respective mutation was found.

^‡^, these mutations arose in the same cell clone;

^†^ reversion.

**Fig 2:**
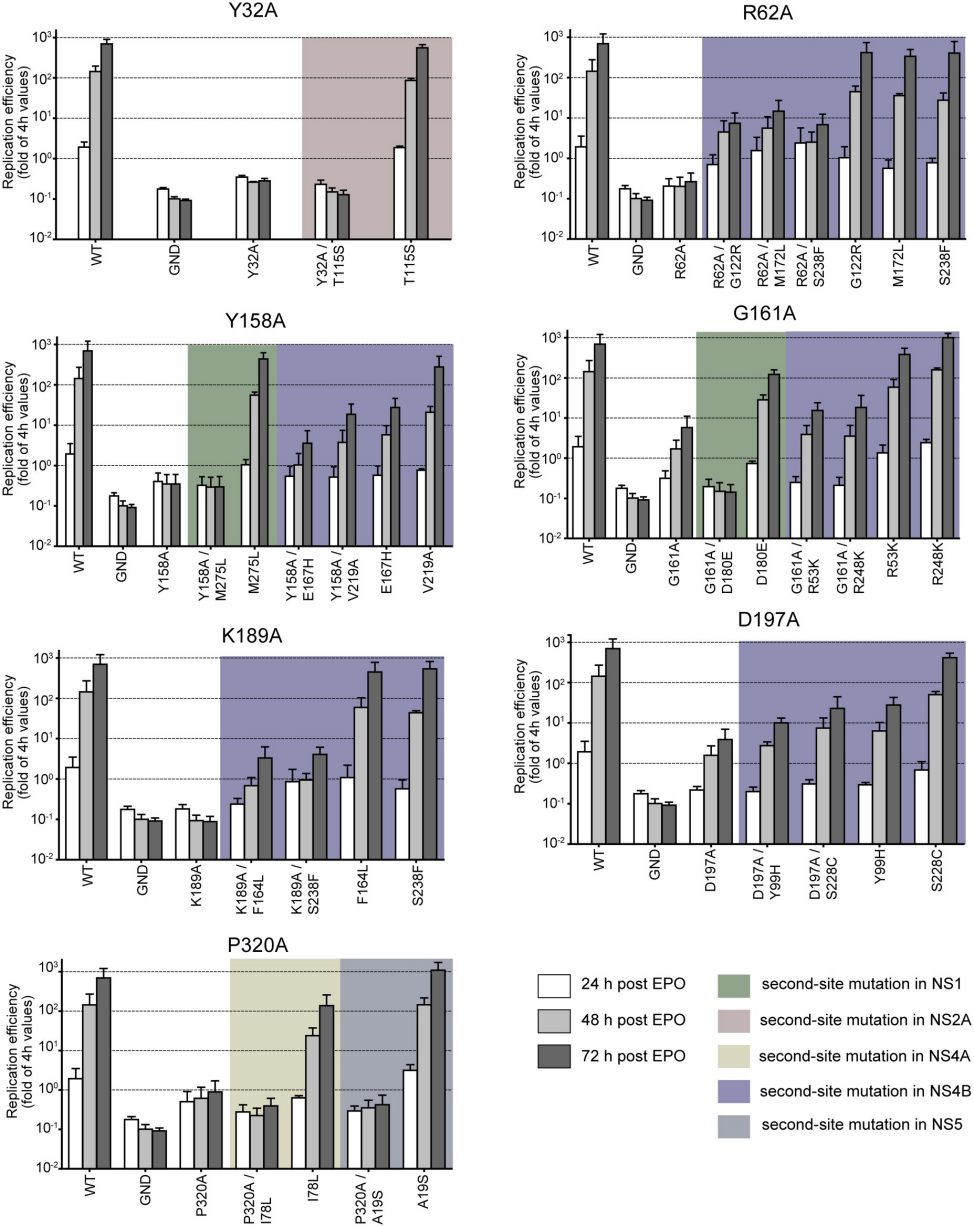
Partial rescue of RNA replication of NS1 mutants by pseudoreversions residing in NS4B. Point mutations in NS1 were inserted alone or together with mutations specified in the bottom of each panel into the full length genome of the DENV Renilla luciferase (RLuc) reporter virus DVR2A. VeroE6 cells were electroporated with *in vitro* transcripts derived from each construct. Cells were lysed at indicated time points after electroporation and RLuc activity was measured to quantify viral RNA replication. For each construct, values were normalized to the 4 h-value to account for differences in transfection efficiency. Results shown are mean values from three independent experiments performed in duplicates with two independent RNA preparations; error bars indicate SD.

With the aim to determine whether those second-site mutations might compensate the replication defect caused by the respective primary NS1 mutation, we inserted each primary mutation together with the respective second-site mutation into a full length DENV-2 genome encoding the *Renilla* luciferase (RLuc) reporter gene (DVR2A), and measured RLuc activity upon transfection of *in vitro* transcribed RNA into VeroE6 cells (Fig 2). Consistent with previous data [17], all primary NS1 mutants were severely impaired in RNA replication, achieving at most 1% of the wildtype replication level at 72 h post-transfection (Fig 2). Importantly, the replication defects of some NS1 mutants was compensated by second-site mutations in NS4B, while tested mutations in other viral proteins did not rescue replication (Fig 2). Although the replication fitness of the double mutants was clearly below wildtype level, we observed a partial restoration in the case of the NS1 mutant R62A by NS4B G122R or M172L or S238F, of the NS1 mutant Y158A by NS4B E167H or V219A, and of the NS1 mutant K189A by NS4B F164L or S238F (Fig 2). In addition, a partial rescue of RNA replication was also found in case of the primary NS1 mutation D197A that was increased by insertion of the pseudoreversion S228C in NS4B. This genetic map supports the proposed role of the NS1—NS4B interaction in the DENV replication cycle [26] and suggests that defects caused by alanine substitutions in NS1 can be compensated, at least in part, by second site mutations in NS4B.

### Establishment of the viral NS1 interactome

**Fig 3:**
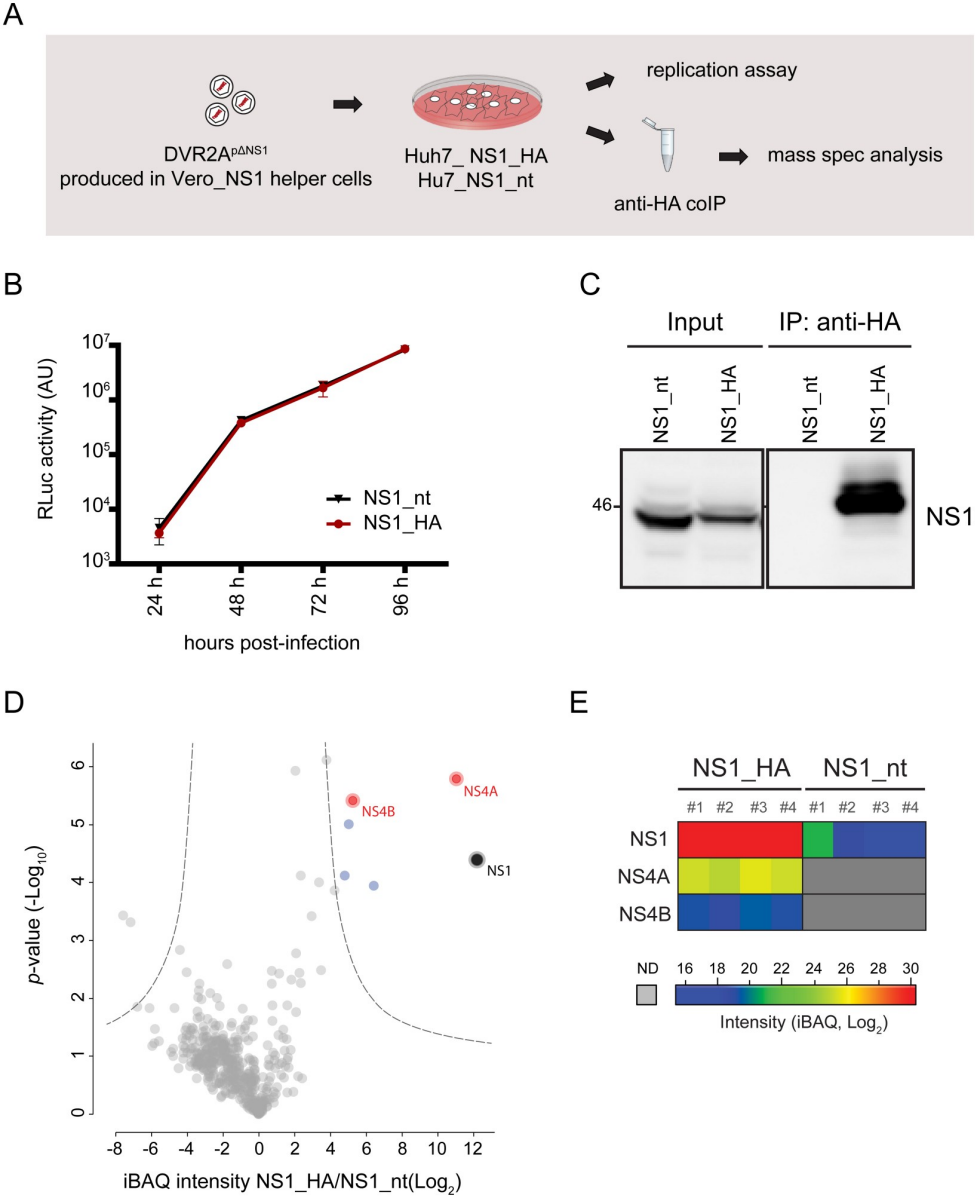
Identification of viral proteins interacting with NS1. (A) Schematic of the experimental approach. Cells stably expressing NS1 were infected with DVR2A containing an in-frame deletion in NS1 (DVR2A^pΔNS1^) and processed to measure viral RNA replication or for affinity purification of HA-tagged NS1 and subsequent analysis of protein complexes by mass spectrometry. (B) DVR2A^pΔNS1^ replication in Huh7 cells stably expressing C-terminally HA-tagged or non-tagged (nt) NS1. Cells were infected with DVR2A^pΔNS1^ (MOI = 1) and lysed at indicated times points post-infection. Viral replication was measured by luciferase assay. (C) Huh7 cells stably expressing NS1_nt or NS1_HA were infected with DVR2A^pΔNS1^ (MOI = 1). Seventy-two hours post infection cells were collected and subjected to HA-specific pull-down. Immune precipitated complexes were analyzed by western blot using a NS1-specific rabbit antiserum. (D) Immune purified complexes prepared as in (C) were subjected to mass spectrometry analysis. Four independent affinity purifications were performed for each bait. Shown is a volcano plot displaying the average degree of enrichment by NS1_HA over NS1_nt (ratio of Intensity-Based Absolute Quantification [iBAQ] protein intensities) and the *P* value (Student’s t-test) for each protein. Significantly enriched proteins are separated from background proteins by a hyperbolic curve (dotted grey line). Viral and host proteins specifically binding to NS1_HA are represented as red and blue dots, respectively. (E) Heat map showing non-imputed log_2_-transformed iBAQ intensities for each individual replicate (see color scale). Only the bait protein and the viral interaction partners are depicted. Gray color represents missing values (not determined [ND]).

Although our forward genetic approach identified NS4B as primary cooperation partner of NS1, we hypothesized that NS1 might promote viral RNA replication not only via association with NS4B, but also via interaction with other DENV proteins. Therefore, we determined the viral NS1 interactome by using affinity purification followed by liquid chromatography and tandem mass spectrometry analysis of captured complexes. To this end we took advantage of a previously reported trans-complementation system [17] allowing functional tagging of NS1 in the context of viral infection in VeroE6 cells. We adapted this system to Huh7 cells because these cells are of human origin and have been used extensively by us and others to study the DENV replication cycle in detail. Huh7 cells stably expressing HA-tagged or non-tagged NS1 were infected with a DENV reporter virus containing an in-frame deletion within NS1 (DVR2A^pΔNS1^) (Fig 3A). In this setting, viral replication could be readily detected at 48 h p.i. demonstrating efficient trans-complementation of NS1 also in Huh7 cells (Fig 3B).

Taking advantage of this system we isolated NS1 by HA-specific precipitation and analyzed captured protein complexes by mass spectrometry. The specificity of the pull-down was confirmed by western blot from samples prepared in parallel experiments (Fig 3C). Samples from cells expressing non-tagged NS1 were used as control to exclude proteins binding non-specifically to the resin. In addition to several host proteins (Fig 3D), NS4A and NS4B were the only potential viral interaction partners of NS1 identified with this approach (Fig 3D and 3E), with the NS1 –NS4B interaction being consistent with our results from the forward genetic screen.

### NS1 interacts with the NS4A-2K-4B precursor, but not with fully processed NS4A or NS4B

**Fig 4:**
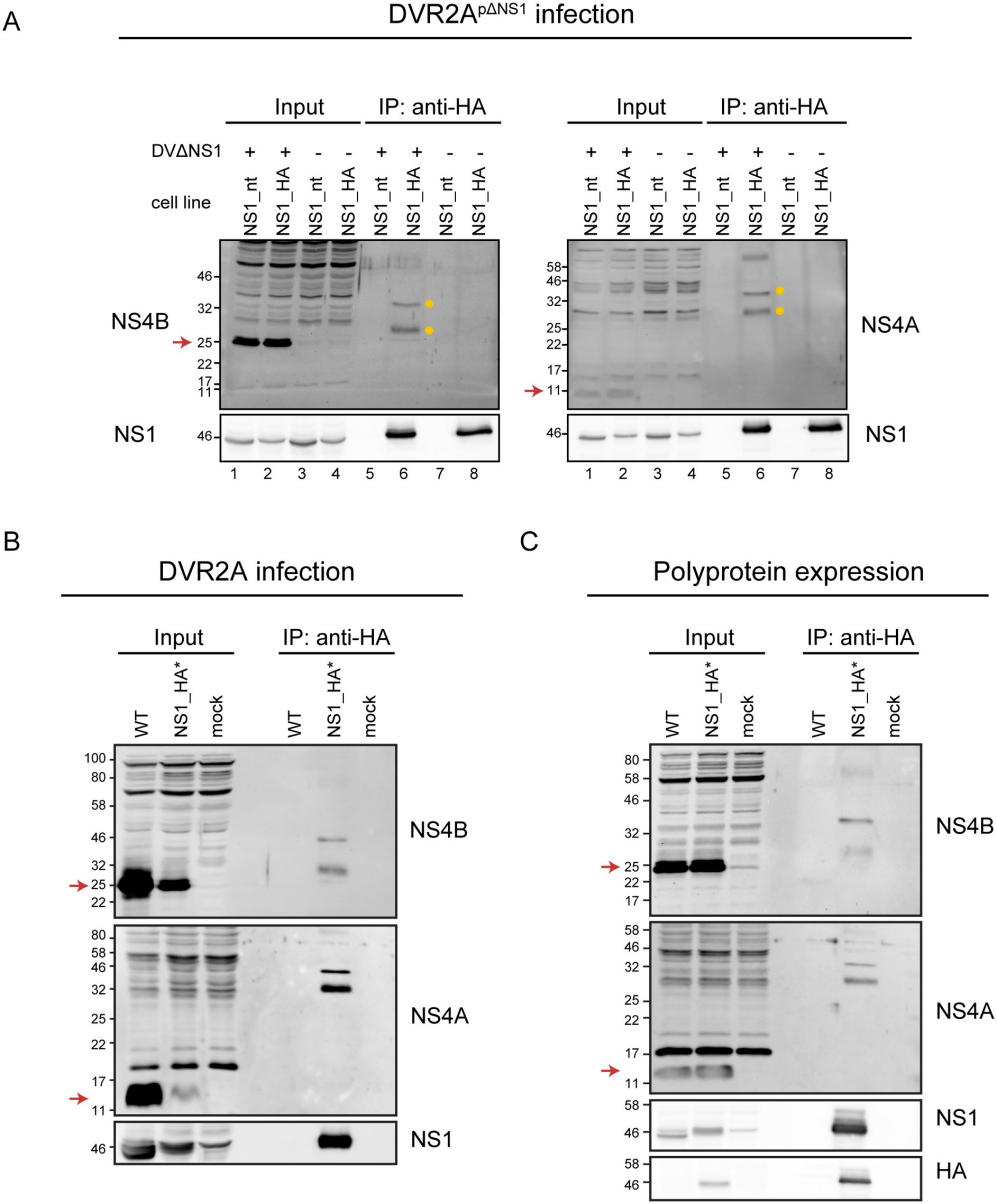
The NS4A-2K-4B precursor polyprotein is the main interaction partner of NS1. (A) Huh7 cells stably expressing carboxy-terminally HA tagged or non-tagged (nt) NS1 were infected with the DVR2A^pΔNS1^ virus (MOI = 1) or mock infected and cell lysates prepared 72 h after infection were subjected to immunoprecipitation (IP) using beads coated with HA-specific antibodies as shown in Fig 3A. Samples were analyzed by western blot using NS4B-, NS4A- or NS1-specific antisera. Mature forms of NS4B and NS4A migrate with an apparent molecular weight of ~25 kDa and ~11 kDa, respectively (indicated by red arrows on the left side of each panel). The NS4A-2K-4B precursor bands migrate at ~30 kDa and ~35 kDa and are highlighted with yellow dots. (B) Huh7 cells were infected with the DVR2A wildtype (WT) or the DVR2A variant encoding an internally HA-tagged NS1 (NS1_HA*) and cell lysates prepared 72 h post infection were subjected to anti-HA immunoprecipitation followed by western blot as described in (A). (C) Huh7-Lunet_T7 cells were transfected with an NS1 to 5 polyprotein expression construct encoding either wildtype or internally HA-tagged NS1 under control of the T7 RNA polymerase promoter. Cells were lysed 20 h post transfection and lysates were subjected to anti HA immunoprecipitation as described in (A).

Focusing our analysis on viral NS1 interaction partners, we confirmed them by evaluating HA-captured protein complexes by immunoblot using NS4B-specific antibodies. As shown in Fig 4A (left panel, lane 1 and 2), NS4B migrates with an apparent molecular weight (MW) of ~25 kDa as a double band with the higher, less pronounced, band likely corresponding to the uncleaved 2K-4B form. Surprisingly, none of these two NS4B species could be detected in HA-NS1 captured immune complexes. Instead, two proteins with apparent molecular weights of ~35 kDa and ~30 kDa, both reacting with the NS4B-specific antibody, were consistently detected (Fig 4A, left panel, lane 6). Both protein species also reacted with a NS4A-specific antibody (Fig 4A, right panel, lane 6), while fully processed NS4A with an apparent MW of ~10 kDa was only visible in total cell lysates from infected cells, but did not co-precipitate with NS1 (Fig 4A, right panel, lane 1 and 2 vs 6). This result suggests that one or both of the 30 and 35 kDa protein species likely corresponds to the uncleaved precursor of NS4B, i.e. NS4A-2K-4B. Notably, this putative precursor protein was highly enriched in HA-NS1 immune captured complexes whereas in lysates of infected cells, mature NS4A and NS4B were the predominant species.

In order to assure that the observed interaction was not a result of NS1 overexpression in *trans*, we inserted the HA tag into NS1 in the context of a full length DENV reporter virus genome (DVR2A-NS1_HA*). Consistent with a previous report [25], epitope-tagged virus was replication competent, although slightly attenuated when compared to the wildtype (S1 Fig). Strikingly, upon HA-specific pulldown the same protein species cross-reacting with NS4A and NS4B specific antibodies were observed (Fig 4B), while no mature form of NS4A or of NS4B could be detected.

To exclude any effect of unequal protein levels caused by the attenuation of the epitope-tagged virus, relative to the wildtype, we expressed HA-tagged NS1 in the context of a NS1 to 5 polyprotein by using a construct encoding all NS proteins under control of the T7 RNA polymerase promoter. Upon expression in Huh7-Lunet_T7 cells [30], the putative NS4A-2K-NS4B precursor was the only protein species enriched in the NS1_HA-specific complexes (Fig 4C). Taken together these results demonstrate that the NS1 –NS4A-2K-4B interaction is not an artifact arising from the individual overexpression of NS1 in *trans* and occurs also in DENV-infected cells.

**Fig 5:**
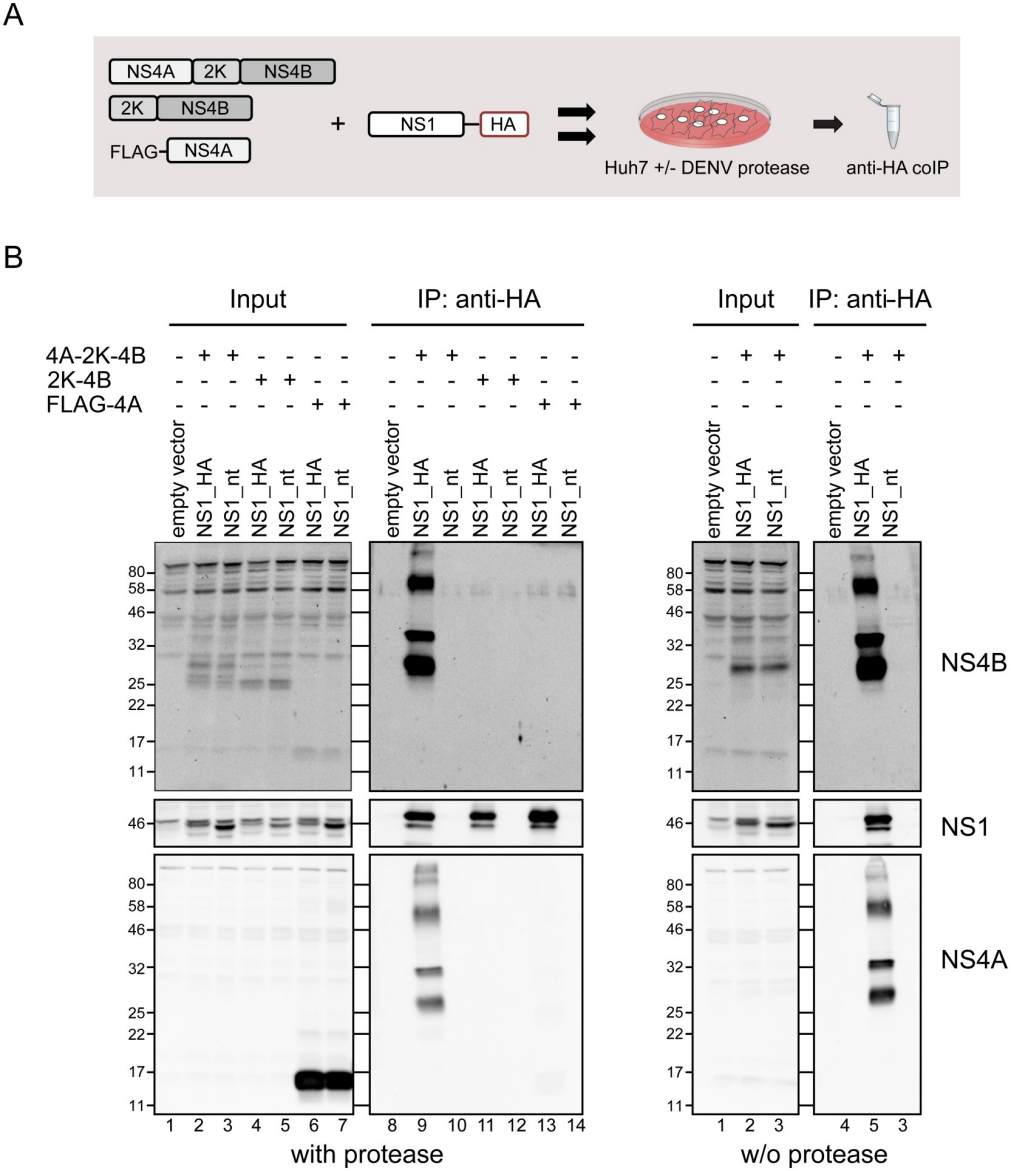
Characterization of the interaction between NS1 and the NS2A-2K-4B cleavage intermediate. (A) Schematic of the experimental approach. (B) Huh7 cells stably expressing the T7 RNA polymerase and DENV NS2B-3 (with protease) or only the T7 polymerase (w/o protease) were co-transfected with plasmids encoding carboxy-terminally HA-tagged or non-tagged NS1 and NS4A and/or NS4B constructs indicated above each lane. Sixteen hours post transfection cell lysates were prepared and subjected to anti-HA immunoprecipitation. Captured protein complexes were analyzed by immunoblotting using rabbit sera reacting with DENV proteins indicated on the right.

Aiming to further characterize the putative NS4A-2K-4B precursor species, we next analyzed the interaction between NS1 and various forms of NS4A and NS4B by using transient expression. To this end, Huh7_T7 cells stably expressing DENV-2 NS2B-3, which is required for polyprotein cleavage, were transfected with equal amounts of HA-tagged NS1 and NS4B or NS4A expression constructs (Fig 5A) and cell lysates were subjected to HA-specific immunoprecipitation. As reported previously [31], *trans* cleavage of the polyprotein by the DENV protease is possible, albeit with limited efficiency, giving rise to three forms of NS4B-containing proteins in lysates of Huh7_T7_NS2B-3 cells transfected with the NS4A-2K-4B construct (Fig 5B, left panel, lane 2 and 3, MW 25–30 kDa). Those 3 forms most likely correspond to the full length NS4A-2K-4B precursor, 2K-4B formed after cleavage by the NS2B-3 protease, and mature NS4B released after additional cleavage by cellular signalase, respectively. Comparable to DENV-infected cells, two protein species with apparent MW of ~30 and ~35 kDa reacting with both NS4A- and NS4B-specific antibodies were detected in HA-NS1 immune purified complexes, together with additional higher molecular weight species (Fig 5B, lower left panel, lane 9). While the theoretical molecular weights for NS4A, NS4B and the NS4A-2K-4B precursor are 16, 27 and 45 kDa, respectively, an abnormal migration pattern of those transmembrane proteins has been observed by us and others with the precursor having an apparent MW of ~30–35 kDa [31–33]. This is consistent with the migration pattern observed here and close to the MW of the precursor reported by others for DENV and other flaviviruses [33–35]. Therefore, we conclude that the lower band detected in the NS1 precipitates corresponds to the monomeric uncleaved NS4A-2K-4B precursor. Importantly, no interaction with NS1 was detected upon co-expression of NS1_HA and 2K-NS4B or NS4A_FLAG (Fig 5B, left panel, lanes 11–14).

To confirm that all protein species observed in NS1_HA immune captured complexes indeed represent the uncleaved NS4A-2K-4B polyprotein, we performed the same experiment in the absence of the DENV protease by using parental Huh7_T7 cells. Under those conditions the NS4A-2K-4B polyprotein should not be processed, because NS2B-3-mediated cleavage at the NS4A—2K site is a prerequisite for signalase-mediated cleavage between 2K and NS4B [31–33,35] As shown in Fig 5B (right panel, lane 5), NS1_HA immune purified complexes from Huh7_T7 cells contained all the NS4-reactive protein species observed in the presence of the DENV protease. Moreover, the same protein pattern was detected by using a commercially available NS4B-specific antibody and after treatment with PNGase (S2 and S3 Figs). These results corroborate that the NS4-reactive proteins with an apparent molecular weight lower than 50 kDa correspond to incompletely processed forms of the NS4A-2K-4B polyprotein, with the higher ~35 kDa band likely representing an incompletely denatured form of the lower, ~30 kDa species. The higher molecular weight species (above 60 kDa) might correspond to lipid-bound NS4 protein species or a heat-stable dimer of the NS4A-2K-4B precursor. The NS4A-2K-4B cleavage intermediate likely contains at least 6 transmembrane helices (Fig 1C) [7,36], which could explain this abnormal migration pattern. In summary, these results show that the NS4A-2K-4B precursor, but not mature NS4A or NS4B, constitutes the main interaction partner of NS1.

### Two residues in the *Wing* connector domain are critical for NS1 interaction with the NS4A-2K-4B cleavage intermediate

**Fig 6:**
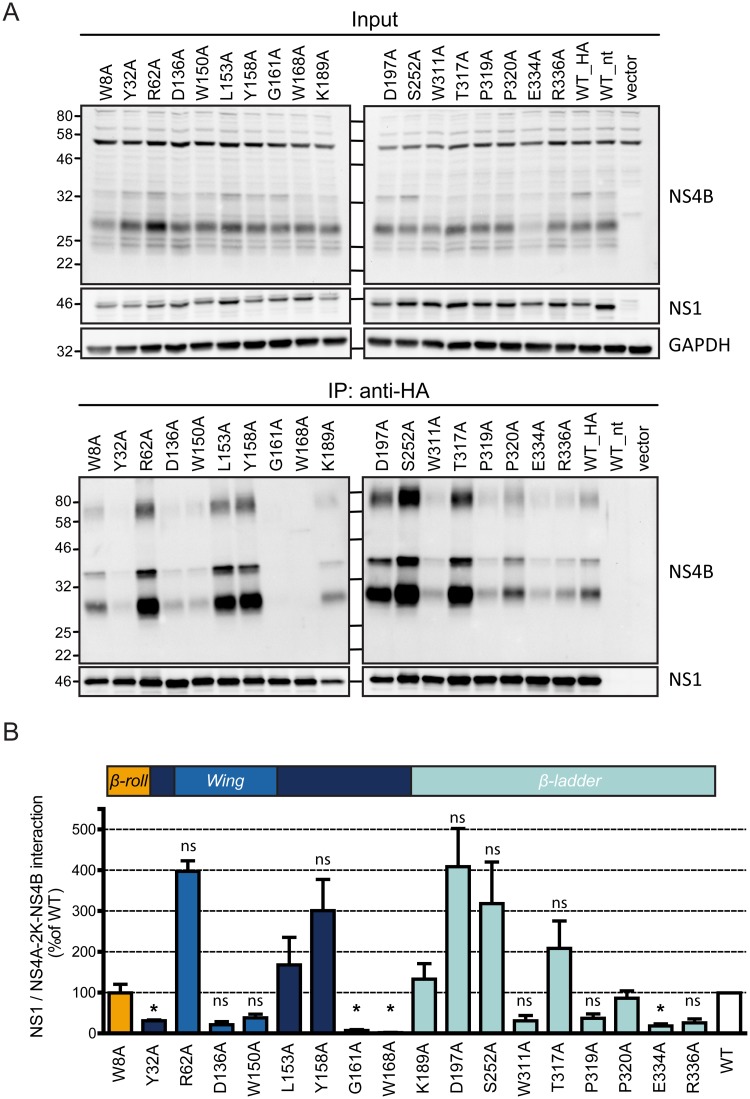
Two residues in the connector region of the *Wing* domain are essential for NS1 interaction with the NS4A-2K-4B precursor. (A) Huh7 cells stably expressing the T7 RNA polymerase and proteolytically active DENV NS2B-3 were co-transfected with constructs encoding HA-tagged wildtype or mutated NS1 and the NS4A-2K-4B polyprotein construct. Cell lysates were processed as described in Fig 4B and immunoblots were probed with NS1- and NS4B-specific antisera. A representative result of four independent experiments is shown. (B) Quantification of NS1 and NS4B-specific signals from all four experiments. In the case of NS4B, the signals of the two bands at ~30 and ~35 kDa were added and used to calculate pull down efficiency. Cells expressing non-tagged NS1 were used to determine the background of the assay that was subtracted from the NS1_HA values. Bars represent the means of the NS4B/NS1 signal ratio, normalized to the wildtype, from four independent experiments. Error bars indicate SEM. *, p<0,002. In all other cases, the difference to the wildtype was not significant (ns).

Having identified an interaction between NS1 and the NS4-2K-4B precursor, we next sought to address the importance of this interaction for viral replication. We predicted that the point mutations in NS1 abrogating the replication might result in loss of binding between NS1 and this cleavage intermediate. To address this hypothesis NS1 point mutants were analyzed in the transient transfection setting described in Fig 5A. As shown in Fig 6, the replication-impairing G161A and W168A mutations resulted in almost complete loss of the interaction with the precursor polyprotein, arguing that the association between NS1 and NS4A-2K-4B is required for RNA replication. Consistently, two other mutations in NS1, Y32A and E334A also reduced the interaction, albeit to a lesser extent. Out of those four mutations decreasing the NS1 –NS4A-2K-4B interaction, second-site mutations in NS4A or NS4B were only identified for the NS1 G161A mutant. These second-site mutations had no impact on NS1 –NS4A-2K-NS4B interaction (S4 Fig), which is consistent with the very low replication rescue provided by those pseudoreversions (maximum 5% of wildtype levels at 72 h post transfection, Fig 2). Several other replication-inactivating NS1 mutations had no statistically significant effect on NS1—4A-2K-4B interaction (summarized in Table 2) suggesting that those NS1 mutants have a replication defect that is independent from the interaction with this cleavage intermediate. In summary, our results identify two residues in the connector region of the *Wing* domain of NS1 that play a crucial role for NS1 interaction with the NS4A-2K-4B precursor and indicate a previously unappreciated importance of this cleavage intermediate for DENV replication.

**Table 2:** Summary of the effects of point mutations in NS1 on NS1—NS4A-2K-4B interaction and NS1 secretion.

Mutation	Domain	NS1—NS4A-2K-4B interaction (fold of WT)	NS1 secretion (fold of WT)
**WT**	**-**	1.00 ± 0.00	1.00 ± 0.00
**W8A**	β-roll	0.99 ± 0.21	0.85 ± 0.18
**Y32A**	Wing (connector)	0.31 ± 0.02	0.91 ± 0.22
**R62A**	Wing (α/β subdomain)	3.97 ± 0.26	0.75 ± 0.16
**D136A**	Wing (α/β subdomain)	0.21 ± 0.08	**0.00 ± 0.02**
**W150A**	Wing (α/β subdomain)	0.38 ± 0.09	0.61 ± 0.05
**L153A**	Wing (connector)	1.67 ± 0.67	0.84 ± 0.15
**Y158A**	Wing (connector)	3.01 ± 0.77	0.99 ± 0.16
**G161A**	Wing (connector)	**0.07 ± 0.02**	0.99 ± 0.16
**W168A**	Wing (connector)	**0.02 ± 0.01**	0.97 ± 0.18
**K189A**	β-ladder	1.33 ± 0.38	0.87 ± 0.11
**D197A**	β-ladder	4.09 ± 0.94	1.09 ± 0.23
**S252A**	β-ladder	3.18 ± 1.02	1.12 ± 0.14
**W311A**	β-ladder	0.31 ± 0.13	**0.03 ± 0.01**
**T317A**	β-ladder	2.08 ± 0.67	0.58 ± 0.06
**P319A**	β-ladder	0.37 ± 0.11	**0.03 ± 0.02**
**P320A**	β-ladder	0.86 ± 0.18	**0.09 ± 0.03**
**E334A**	β-ladder	0.18 ± 0.05	**0.02 ± 0.02**
**R336A**	β-ladder	0.26 ± 0.10	**0.03 ± 0.02**

**Fig 7:**
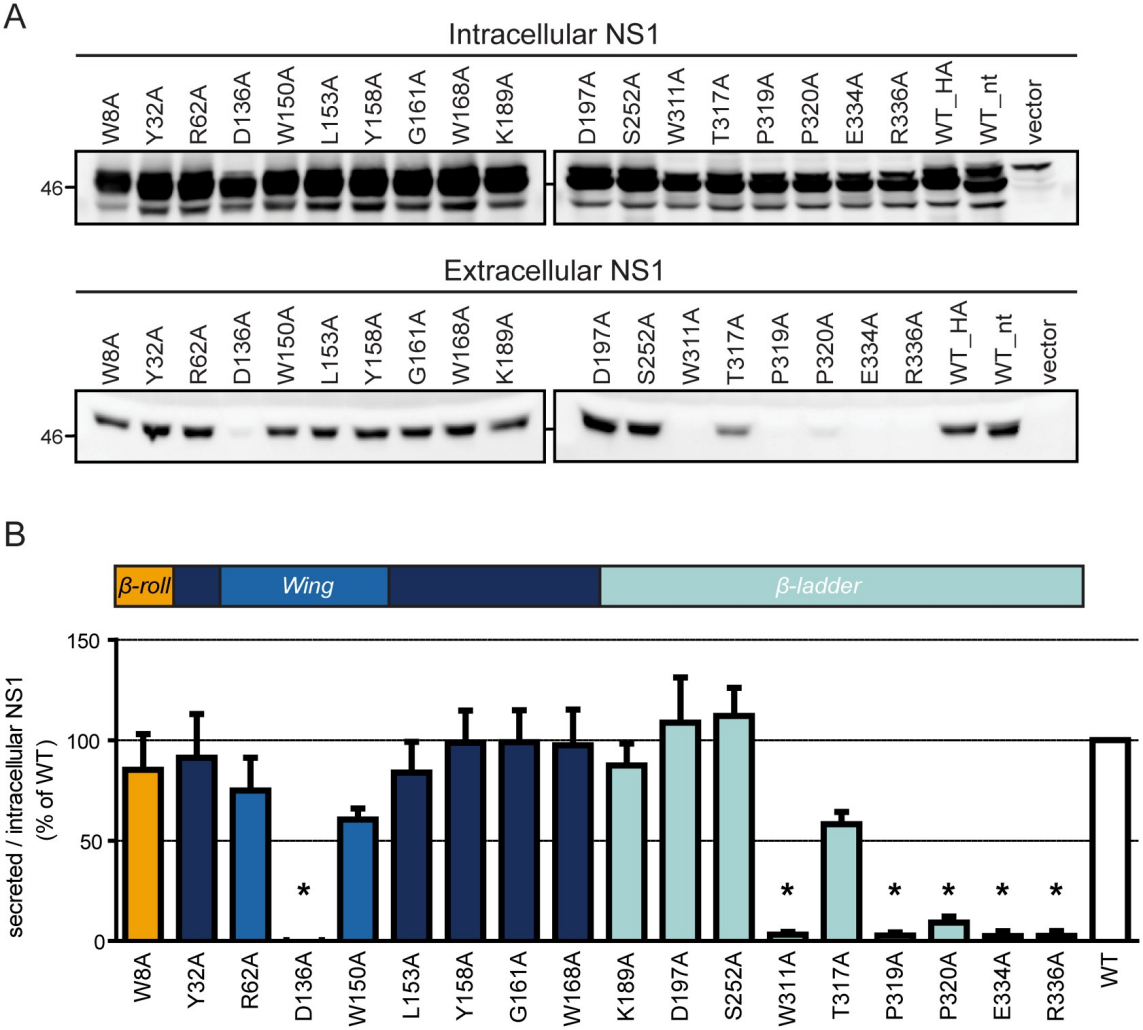
A cluster of conserved amino acid residues in the carboxy-terminal *β*-*ladder* of NS1 is essential for NS1 secretion. (A) Huh7 cells stably expressing the T7 RNA polymerase were transfected with plasmids encoding HA-tagged NS1 that contained point mutations specified above each lane. Sixteen hours later cell culture supernatants were collected and cleared by centrifugation. Equal volumes of supernatants and cell lysates were analyzed by immunoblotting using an NS1-specific antiserum. (B) Quantification of NS1 signals from (A). The bars are means of the ratio of secreted to intracellular NS1, normalized to the wildtype, from three independent experiments. Error bars indicate SEM. *, p<0,002. In all other cases, the difference to the wildtype was not significant.

Mutations in NS1 impairing NS4A-2K-4B precursor binding (below 0.1 of WT) and NS1 secretion (below 0.1 of WT) are highlighted in light and dark gray, respectively. Data are mean from at least 3 independent experiments, shown as fold of WT ± SEM. Data correspond to Figs 6 and 7.

### Highly conserved residues in the carboxy-terminal region of NS1 are critical for NS1 secretion

Earlier studies suggested that mutations within the connector region of the *Wing* domain might weaken NS1 association with ER membranes [37], potentially perturbing its secretion. To ensure that the observed loss of interaction with the precursor was not due to increased NS1 secretion, we examined the secretion efficiency for each NS1 mutant. To this end, cells that had been transfected with constructs encoding for various NS1 mutants, and corresponding culture supernatants, were assessed by quantitative western blot (Fig 7). Of note, the two mutations completely blocking the NS1 –NS4A-2K-4B interaction, i.e. G161A and W168A, did not affect NS1 secretion, indicating that the effect of these alanine substitutions on precursor binding was not due to lower intracellular abundance of NS1 as a result of enhanced NS1 release. Interestingly, several other point mutations, mostly residing in the carboxy-terminal region of the β-ladder domain, i.e. W311A, P319A, E334A and R336A almost completely blocked NS1 secretion (Fig 7). While this could be due to structural defects possibly resulting in ER-associated degradation of these NS1 variants, intracellular abundance of these proteins was not strongly affected, arguing that they were not targeted for degradation because of misfolding or destabilization. Instead, they might have a defect in self-interaction or routing to the secretory pathway.

### Replication-inactivating mutations in NS1 do not affect polyprotein cleavage

**Fig 8:**
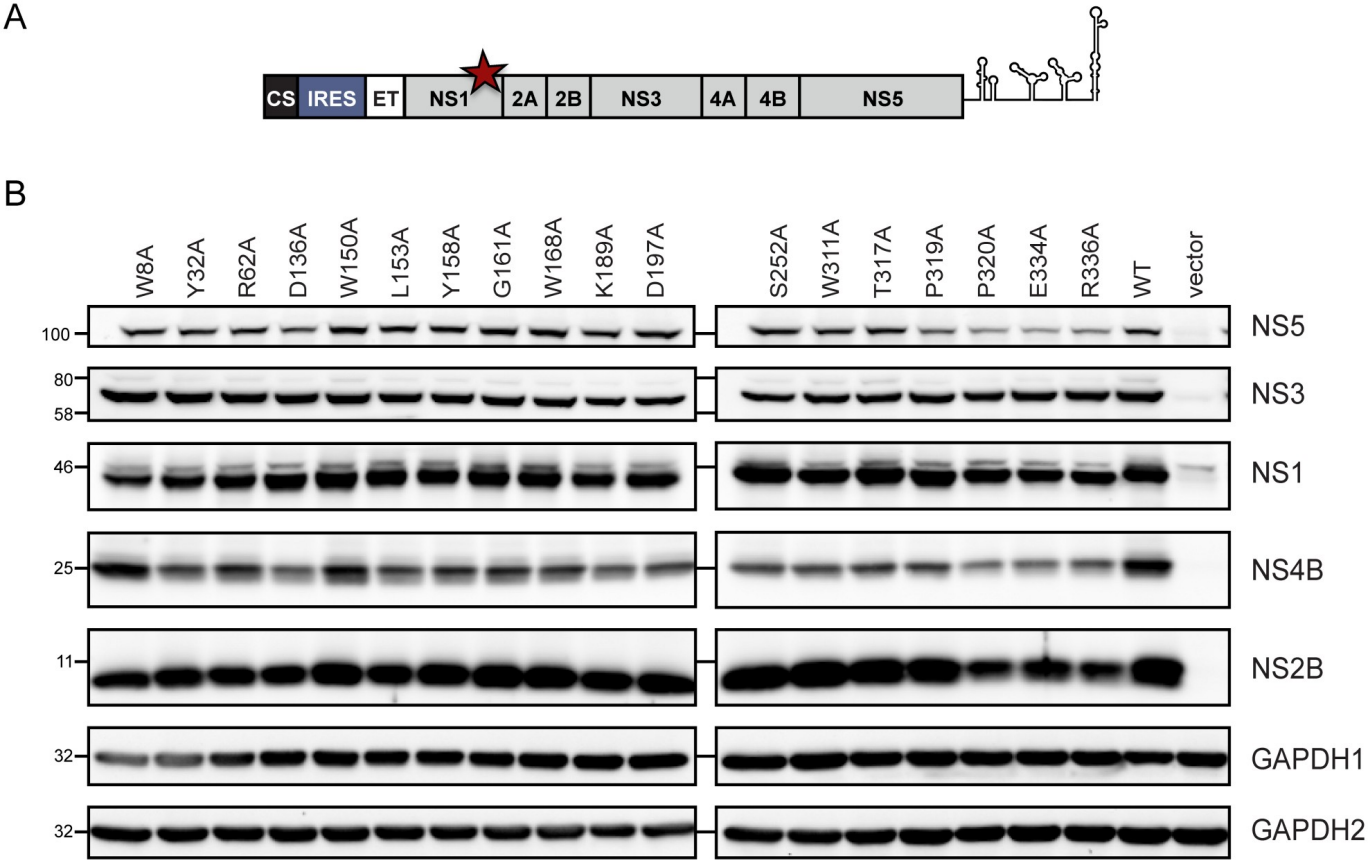
Replication-blocking mutations in NS1 do not impact polyprotein processing. (A) A schematic of the expression cassette used to produce the DENV-2 polyprotein. (B) Huh7-Lunet T7 cells were transfected with the polyprotein expression constructs harboring mutations in NS1 specified above each lane. Eighteen hours post transfection cells were lysed and processed for western blot by using antisera specific to DENV proteins given in the right of each panel. GAPDH was used as loading control. GAPDH 1 corresponds to membranes probed for NS1, NS2B and NS5; GAPDH 2 for membranes probed with NS3 and NS4B. A representative result of three independent experiments is shown.

We next asked whether the alanine substitutions we had inserted into NS1 might have an impact on DENV polyprotein processing, especially the production of fully cleaved NS4B. Therefore, we inserted the alanine substitutions into an expression construct encoding the complete DENV polyprotein that was expressed by a T7 promoter-based system to allow replication-independent protein production (Fig 8A). Huh7-Lunet_T7 cells were transfected with these constructs and 18 h later cells were lysed and NS1, NS2B, NS3, NS4B and NS5 expression was detected by western blot. None of the point mutations in NS1 had an obvious impact on polyprotein processing (Fig 8B), at least under steady state conditions, demonstrating that the inhibitory effects exerted by these mutations (block of RNA replication, NS4A-2K-4B interaction and NS1 secretion) did not result from improper polyprotein cleavage.

### Formation of vesicle packets is independent from interaction between NS1 and the NS4A-2K-4B cleavage intermediate

**Fig 9:**
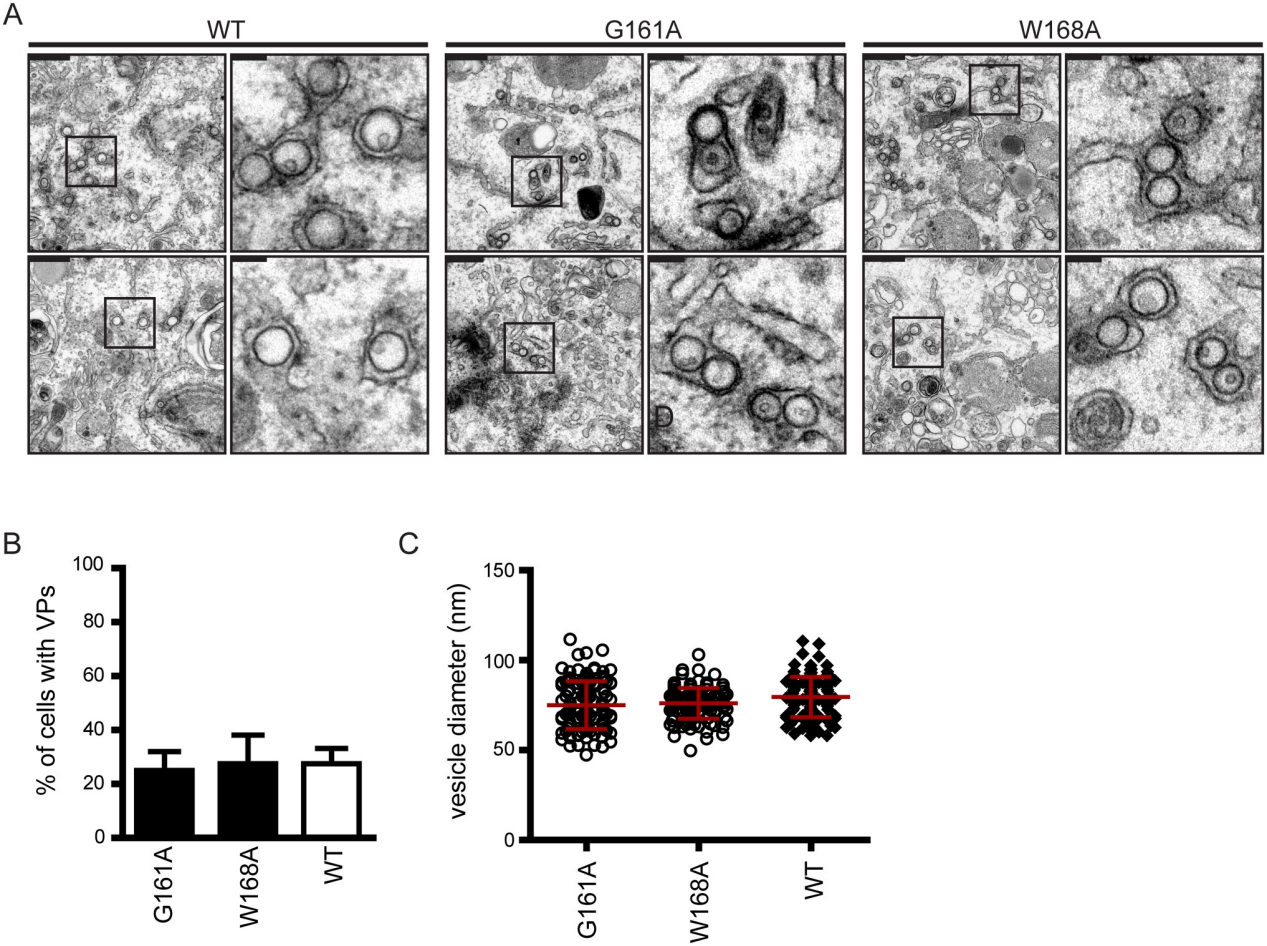
Formation of DENV vesicle packets is independent from the interaction between NS1 and the NS4A-2K-4B precursor. Huh7-Lunet T7 cells were transfected with the DENV polyprotein expression construct shown in Fig 8A and 18 h later, cells were fixed and processed for transmission electron microscopy. (A) Representative images of membrane invaginations observed in cells transfected with the wildtype (WT) polyprotein, the G161A or the W168A mutant, respectively. Scale bars (upper left of each panel) correspond to 500 nm in the overview and 100 nm in the cropped sections that are indicated with black rectangles in the overviews. (B) Quantification of the number of cells containing VPs. Results show the mean of two independent experiments, counting at least 20 cells per construct and experiment. The error bars indicate SD. Transfection efficiency as determined by immunofluorescence was ~45%. (C) Quantitation of the vesicle diameter in cells transfected with WT, G161A or W168A polyprotein construct. Scatter plots indicate the diameter of >100 vesicles from two independent experiments; horizontal lines indicate means and error bars indicate SD.

One of the proposed mechanisms by which NS1 supports DENV RNA replication is the formation of membranous replication organelles, i.e. the VPs. Since both NS4A and NS4B are transmembrane proteins with membrane remodeling properties [7,33], interaction between NS1 and the NS4A-2K-4B precursor might be important for establishment or stabilization of those membrane structures. Therefore, we wanted to determine whether the two mutations abrogating NS1 interaction with this cleavage intermediate (G161A and W168A) also block VP formation. Thus far, the biogenesis of these membranous structures could only be studied in systems supporting viral replication, making it impossible to determine the impact of replication-impairing mutations on VP formation. To overcome this limitation, we employed a polyprotein expression approach (Fig 9A). In this system, the sole expression of the DENV polyprotein in Huh7-Lunet_T7 cells resulted in the formation of membrane invaginations that closely resemble VPs observed during DENV infection (Fig 9A, left panel) in ~25% of the cells (Fig 9B). The diameter of the vesicles was ~75 nm (Fig 9C), which is comparable to VP diameter in infected cells [4].

Taking advantage of this system, we next inserted the G161A or W168A mutations into NS1 of this expression construct and determined abundance and morphology of vesicle formation (Fig 9). Interestingly, none of the mutations had an effect on number or size of the vesicles, demonstrating that the NS1—NS4A-2K-4B interaction is not involved in VP formation.

### NS1 is required for formation of VPs

**Fig 10:**
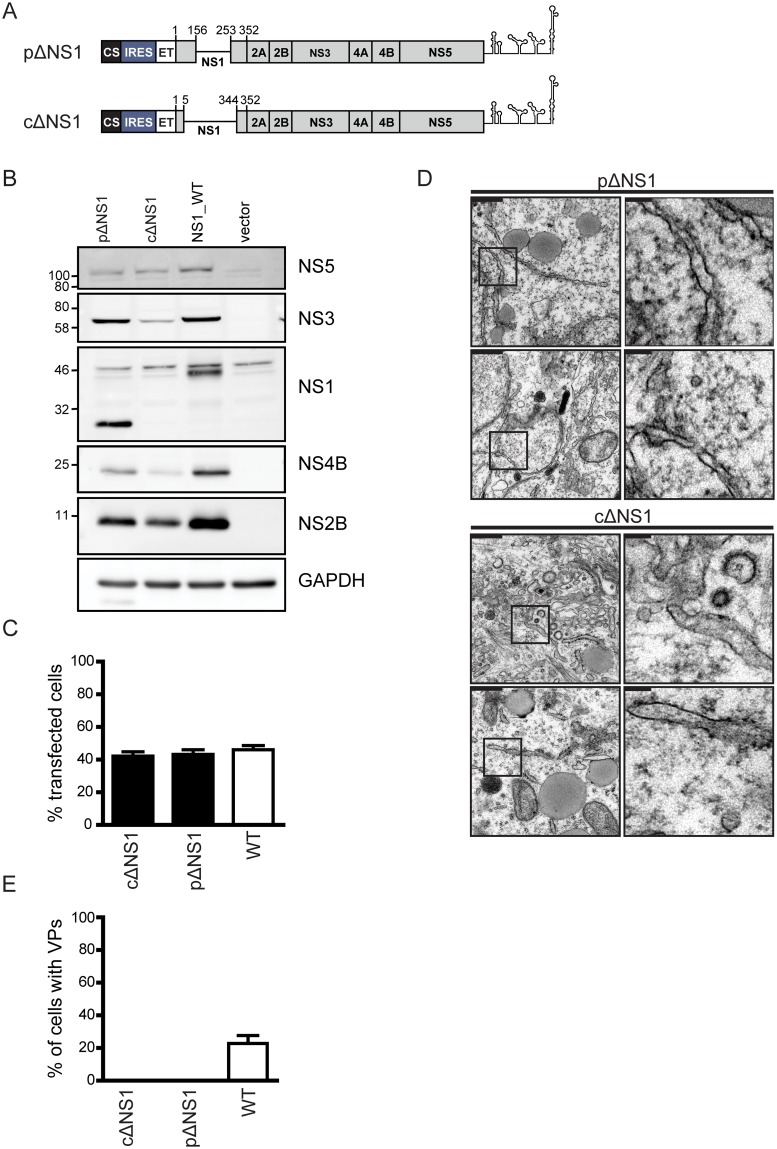
NS1 is essential for the formation of vesicle packets. (A) Schematic representation of the expression constructs containing a partial deletion in NS1 (pΔNS1) or lacking NS1 completely (cΔNS1). Huh7-Lunet_T7 cells were transfected with pΔNS1, cΔNS1 or the wildtype (WT) construct, fixed 18 h post transfection and processed for electron microscopy, western blot or immunofluorescence. (B) Western blot of cell lysates prepared 18 h post transfection and analyzed by using antibodies indicated on the right. A representative experiment of three repetitions is shown. (C) Transfection efficiency of Huh7-Lunet_T7 cells as determined by immunofluorescence. Data are the mean from three independent experiments using two independent plasmid preparations and counting each time at least 200 cells per sample. The error bars indicate the SEM. (D) Representative transmission electron microscopy images of Huh7-Lunet_T7 cells transfected with expression constructs containing a partial or complete NS1 deletion. Scale bars (upper left of each panel) correspond to 500 nm in the overview and 100 nm in the cropped sections that are indicated with black rectangles in the overviews. (E) Quantification of the EM analysis. The percentage of cells with VPs is shown. Note the absence of regular VPs in cells expressing either NS1 deletion mutant. Data are based on 4 independent experiments, using two independent plasmid preparations and counting at least 20 cells per construct and per repetition.

We next asked whether NS1 is at all required for the biogenesis of VPs. To address this question, we used two polyprotein expression constructs. The first contained an in-frame deletion of 97 codons in the NS1 coding region that can be rescued by ectopic expression of NS1 in the context of DENV infection (partial deletion of NS1, pΔNS1) [17], and the second a complete deletion of NS1 (cΔNS1) (Fig 10A). In the cΔNS1 construct the first 5 and last 8 amino acid residues of NS1 were retained to ensure proper polyprotein insertion into the ER membrane and processing, respectively [38]. Huh7-Lunet_T7 cells were transfected with either mutant or the wildtype polyprotein expression constructs and protein expression was determined by western blot (Fig 10B). While viral protein abundance was slightly lower in the case of the two NS1 deletion mutants, the ratio between the viral proteins was similar to the wildtype. Importantly, transfection efficiency was comparable (~40% as determined by immunofluorescence; Fig 10C) and the subcellular distribution of NS4B and NS3 was not affected by the NS1 deletions (S5 Fig). Analysis of the cells by transmission electron microscopy (EM) revealed that both NS1 deletions abrogated the formation of VPs (Fig 10D and 10E) showing that NS1 is required for VP formation.

**Fig 11:**
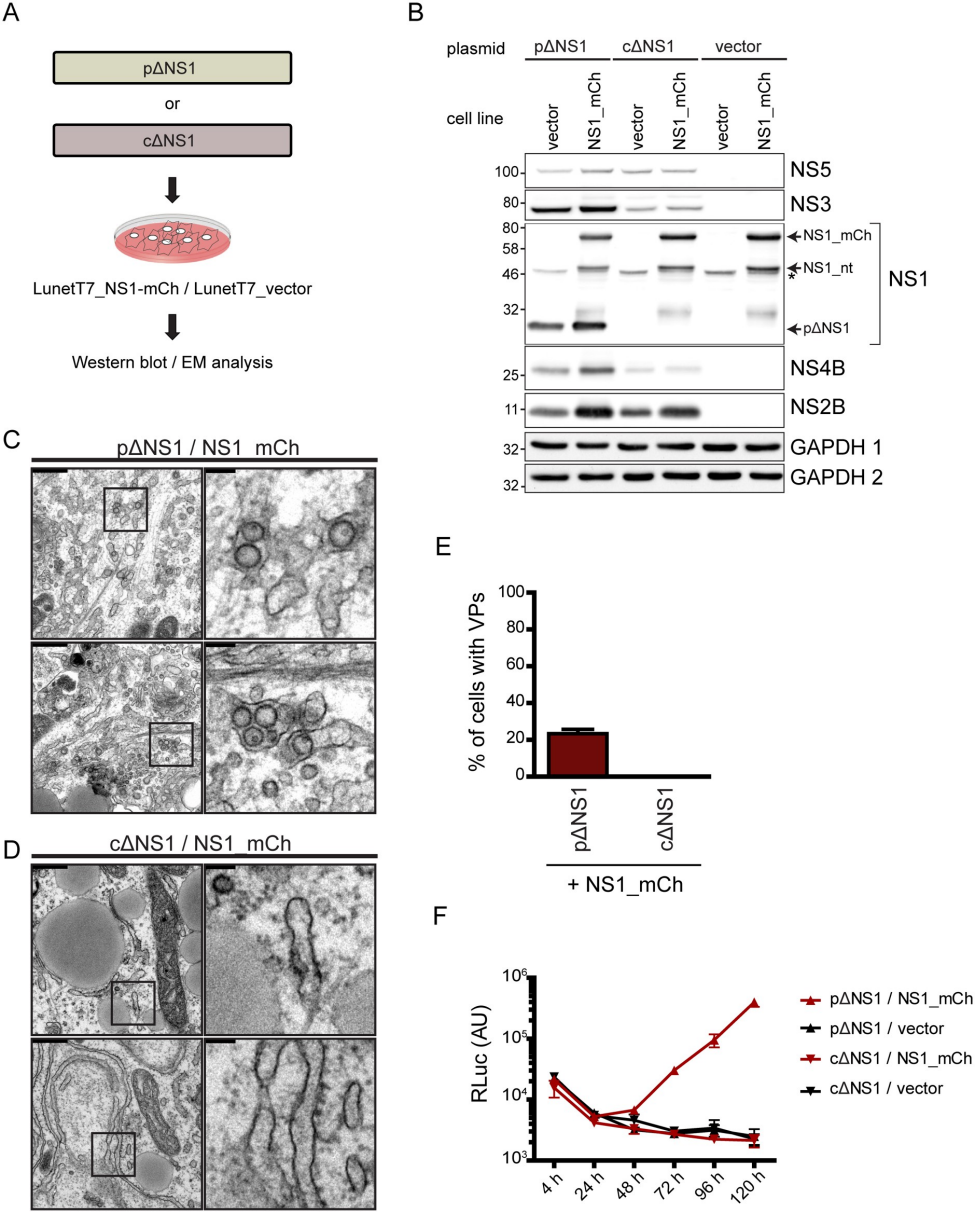
Rescue of vesicle packet formation by NS1 provided in *trans*. (A) Huh7-Lunet_T7 cells stably expressing NS1 with a carboxy-terminal mCherry tag (NS1_mCh) or the empty vector were transfected with pΔNS1, cΔNS1 or the wildtype (WT) polyprotein expression constructs. Cells were harvested 18 h post-transfection and processed for western blot and EM analysis. (B) Expression of DENV proteins in transfected Huh7-Lunet_T7 cells. Black arrows indicate NS1 variants; the star indicates an unspecific background signal. GAPDH was used as loading control. GAPDH 1 corresponds to membranes probed for NS1, NS2B and NS5; GAPDH 2 for membranes probed with NS3 and NS4B. (C) Representative electron micrographs of VPs in cells expressing NS1-mCh and transfected with the pΔNS1 polyprotein expression construct. (D) Absence of VPs in NS1_mCh expressing cells transfected with the cΔNS1 polyprotein expression construct. Scale bars (upper left of each panel) in (C) and (D) correspond to 500 nm in the overview and 100 nm in the cropped sections that are indicated with black rectangles in the overviews. (E) Quantification of the number of cells containing VPs. Data are mean from 2 independent experiments, counting at least 20 cells per condition; error bar indicates SD. (F) Replication of DVR2A containing a partial (pΔNS1) or complete (cΔNS1) deletion of NS1 in Huh-7-Lunet_T7 cells expressing NS1_mCh or control cells (stably transduced with the empty vector). Cells were transfected with *in vitro* transcribed RNA derived from the respective construct by electroporation, lysed at indicated time points post transfection and RLuc activity was measured to quantify viral RNA replication. Results shown are mean values from two independent experiments performed in triplicates; error bars indicate SEM.

Next, we sought to determine whether expression of NS1 in *trans* can rescue the VP defect caused by the deletions within this protein. To this end, we transfected Huh7-Lunet_T7 cells stably expressing mCherry-tagged NS1 (LunetT7_NS1-mCh) with the pΔNS1 or cΔNS1 polyprotein expression construct and assessed VP formation (Fig 11A). Production of the viral proteins in transfected cells was confirmed by western blot (Fig 11B). Of note, the defect of VP formation caused by the partial deletion in NS1 could be restored by expression of NS1 in *trans* (Fig 11C and 11E). By contrast, no such rescue could be observed in cells expressing the polyprotein with the complete deletion in NS1 (Fig 11D and 11E). Remarkably, rescue of VP formation by trans-complementation with NS1 correlated with rescue of viral replication (Fig 11F). While both the partial and the complete NS1 deletion abrogated DENV RNA replication, the provision of NS1 in *trans* rescued the replication of the partial NS1 deletion, but not of the complete NS1 deletion (Fig 11F).

In summary, these results demonstrate that NS1 is indispensable for proper formation of the DENV replication organelle, but this function is not related to interaction of NS1 with the NS4A-2K-4B precursor. The fact that the complete NS1 deletion cannot be rescued by trans-complementation argues that NS1 contains both *cis*- and *trans*-acting determinants involved in VP formation.

## Discussion

Despite intensive research efforts the role of NS1 in the flavivirus replication cycle remains elusive. NS1 is not involved in viral entry or RNA translation but is essential for RNA replication. This observation has originally been made during studies conducted with the yellow fever virus (YFV), and later confirmed with several other flaviviruses [16,17,19,26,39,40]. It is thought that NS1 is required for negative RNA strand synthesis [16,19], but the molecular mechanism is not known.

**Fig 12:**
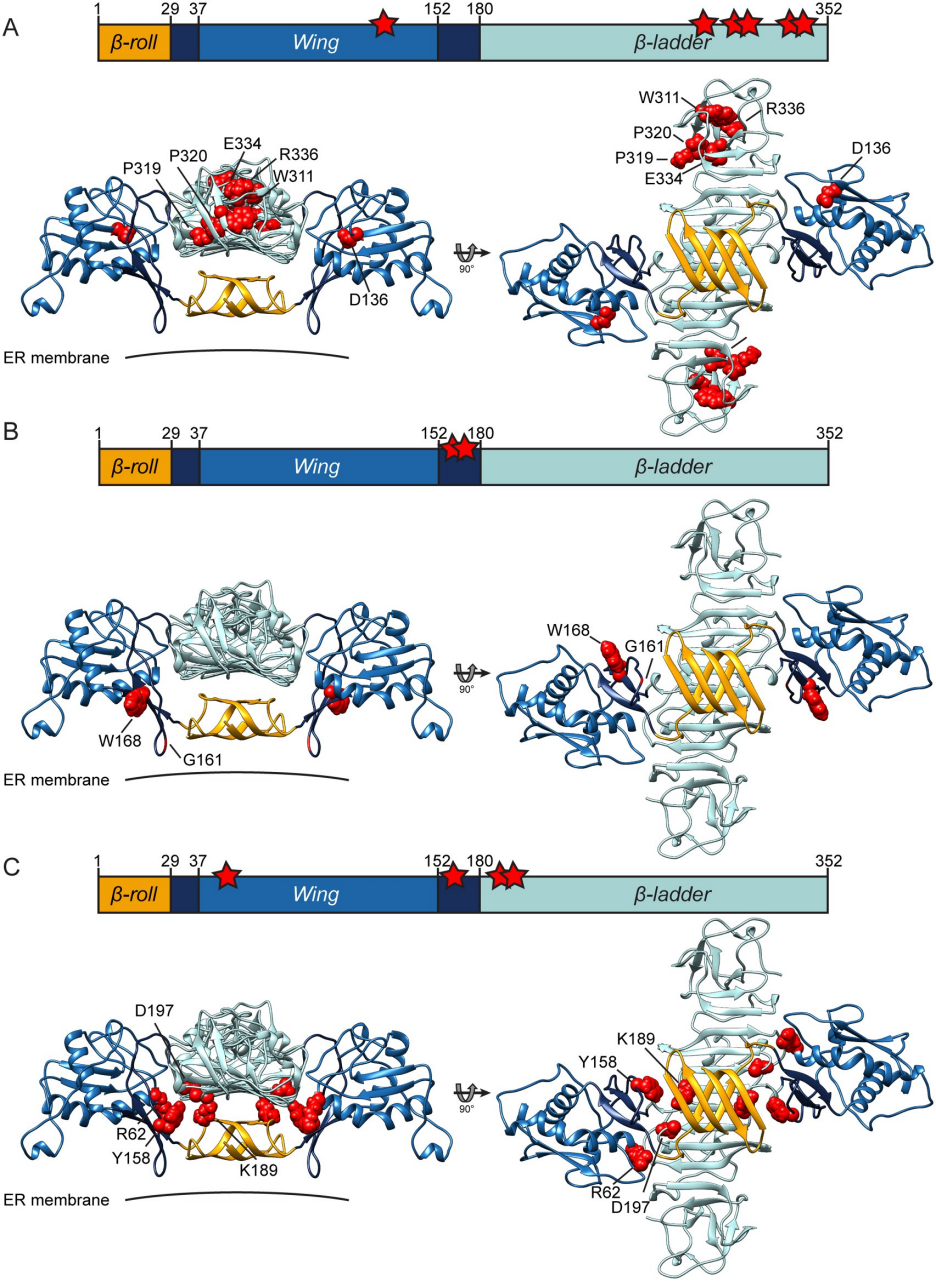
Overview of amino acid residues in NS1 required for NS1 secretion, interaction with the NS4A-2K-4B precursor and RNA replication. Replication-impairing mutations that (A) abolish NS1 secretion, (B) abrogate binding between NS1 and the NS4A-2K-4B precursor, and (C) can be complemented by second site mutations in NS4B. Upper panels show the linear map of NS1 with mutated residues indicated by red stars; bottom panels show the homology model of the 3D structure of NS1 based on PDB entries 4O6B and 5K6K as described in Fig 1(A) with mutated residues shown as van der Waal spheres in red. *Wing*, *β*-*ladder* and *β*-*roll* domains are shown in blue, turquoise and orange, respectively. Connector subdomains in *Wing* domain are shown in dark blue.

In the present study we employed reverse and forward genetic screening, combined with biochemical assays, to identify domains and regions within NS1 that contribute to its role in DENV RNA replication as well as NS1 secretion. With respect to the latter, we observed a clustering of secretion-inhibiting mutations in the highly conserved carboxy-terminal region of the *β-ladder*, which contains the almost invariant MEIRP motif comprising amino acid residues 333–337 (Fig 12A) [41]. Intracellular retention of these mutants, which are also replication deficient, might result from improper NS1 self-interaction, or altered trafficking due to loss of interaction with cellular transport proteins. Interestingly, the carboxy-terminal tip of the *β-ladder* domain contains multiple epitopes recognized by NS1-specific antibodies, including some that cross-react with cellular surface proteins [23,41,42]. Hence, this region might represent an attractive drug target, offering the possibility to block viral replication, NS1 secretion and the induction of antibodies possibly contributing to DENV pathogenesis.

The results of our genetic studies provide strong evidence for an interaction between NS1 and NS4B, or NS4B-containing cleavage intermediates. This conclusion is derived from the observation that replication-inactivating mutations in NS1 can be rescued, at least in part, by pseudoreversions residing in NS4B. This genetic NS1 –NS4B association was corroborated by the viral NS1 proteome identifying NS4B and NS4A as predominant interaction partners of NS1. However, further characterization of the NS1—NS4B association revealed that the main interaction partner of NS1 is the NS4A-2K-4B precursor. This interaction, which has so far not been reported, was consistently detected in complexes isolated from DENV-infected cells and in different expression-based approaches. The NS4A-2K-4B intermediate has been previously detected in YFV-infected cells using pulse-chase experiments, where it was found to be processed post-translationally with a half-life of ~10 min [34]. A subsequent study confirmed the production of NS4A-2K-4B also in the course of DENV infection [35], however its function in virus replication has not been appreciated so far. Based on our findings, and on the high enrichment of NS4A-2K-4B in the NS1-associated protein complexes, we conclude that delayed cleavage of this polyprotein fragment plays an important role for interaction with NS1 as well as RNA replication. Such regulated cleavage has been described for several other positive-strand RNA viruses and also in those cases it critically determines RNA replication [43–45]. For instance, for the related HCV it was found that constitutive cleavage between NS4B and NS5A by insertion of an IRES at the cleavage site completely abrogates RNA replication [43]. Likewise, in the case of alphaviruses processing intermediates and corresponding mature forms were found to play distinct roles in negative and positive RNA strand synthesis, respectively [44]. The importance of various polyprotein precursors is also well established for picornaviruses and their functions include genome circularization [46], modulation of enzymatic activities and modification of cellular membranes (reviewed in [47]).

We speculate that delayed cleavage of DENV NS4A-2K-4B might be required for proper membrane association of NS4A or NS4B, maturation of these proteins e.g. by post-translational modification [48] or the formation of dimeric or oligomeric complexes. Although the exact mechanism remains to be determined, it is tempting to speculate that NS1 binding to NS4A-2K-4B might regulate its cleavage.

Two replication-impairing point mutations in NS1, G161A and W168A, almost completely abrogated NS4A-2K-4B precursor binding, corroborating the specificity of the observed interaction and its critical role in RNA replication. These mutants were stable and efficiently secreted, demonstrating that the loss of interaction was not due to general defects in protein structure, degradation or enhanced NS1 release. Moreover, both mutants were able to form VPs when expressed in the context of the NS1 to 5 DENV polyprotein, implying that subcellular localization and membrane association of NS1 were not affected by these substitutions. Residue G161 localizes to the so called “*greasy finger*” loop within the *Wing* domain of NS1, which has been proposed to mediate NS1 interaction with the ER membrane [23] (Fig 12B). While it might appear surprising that a glycine to alanine substitution had such drastic effects on viral replication and protein-protein interaction, the G159-X-G161 motif is absolutely conserved in flaviviruses [17,49], which is indicative of a critical function of this region in the viral replication cycle. The aromatic amino acid residue at position 168 is also invariant and resides on the NS1—ER membrane interface, contributing to the formation of a hydrophobic “inner face” of the NS1 dimer (Fig 12B). While several other mutants, most notably Y32A and E334A, also showed a trend towards reduced NS1 –precursor interaction (Fig 6 and Table 2), their impact was much weaker compared to the G161A and W168A substitutions. We therefore assume that replication impairment caused by the Y32A and E334A substitutions is due to defects other than impaired interaction with NS4A-2K-4B. This is also very likely for mutations in the C-terminal region that result in loss of NS1 secretion. Of note, neither the mutation affecting residue W8, which resides in the *β-roll* domain and is assumed to be directly involved in NS1—membrane interaction [50], nor the mutation affecting Y158 directly adjacent to the *greasy finger* (S6 Fig) abrogated NS1 –cleavage intermediate binding. This result further suggests that the phenotype caused by G161A and W168A is not due to general alteration of the NS1—membrane interaction, but rather to abrogation of specific contact sites between NS1 and the NS4A-2K-4B cleavage intermediate that are formed by the two residues.

Four of the primary NS1 mutations (R62A, Y158A, K189A and D197A), all residing in the membrane proximal region of the NS1 dimer (Fig 12C), could be compensated by pseudoreversions in NS4B. These primary mutations did not affect NS4A-2K-4B precursor binding, indicating that the observed phenotype was not related to this interaction. Interestingly, all of the pseudoreversions restoring replication were found in the putative transmembrane domains of NS4B (Fig 1C). Given this localization, the primary mutations might impair the membrane remodeling activity of NS1 that might be restored by structural changes in NS4B, consistent with its ability to alter ER membranes as reported for WNV [51]. However, the rescue was only partial suggesting that additional defects, which cannot be restored by NS4B might be caused by these alanine substitutions in NS1.

DENV replication most likely occurs within invaginations in the ER membrane designated VPs [4]. Owing to the lack of systems to study VP biogenesis in the absence of viral replication, little is known about viral and cellular factors involved in their biogenesis [9]. By using transient expression of a DENV polyprotein we were able to analyze VP formation with replication-deficient NS1 mutants. The following important conclusions can be drawn from our EM-based studies: (i) NS1 is essential for the establishment of the membranous DENV replication organelle; (ii) this function is not related to NS1 interaction with NS4A-2K-4B, because mutations disrupting this interaction did not affect VP formation; (iii) at least part of NS1 acts in *cis* to allow for VP formation as deduced from the observation that only the partial, but not the complete NS1 deletion could be rescued by *trans*-complementation. Therefore, NS1 plays a more sophisticated role in viral RNA amplification that goes beyond the structuring of replication complex formation as often proposed [21,52]. Since the G161A and the W168A mutation impaired viral replication without affecting VP formation, we conclude that NS1 has at least two distinct functions, i.e. the assembly of the membranous replication organelle and the generation of an active replicase that catalyzes the amplification of the viral RNA, the latter steps possibly linked to the interaction of NS1 with the NS4A-2K-4B cleavage intermediate. Of note, Akey et al. reported that mutations targeting residues 159–161 of NS1 were deleterious to viral RNA replication, but did not affect the ability of NS1 to remodel liposomes [23]. This result is in consistent with our notion that this region in NS1 is involved in an RNA replication-relevant process that is independent from the formation of VPs.

Studies conducted with YFV and West Nile virus revealed that trans-complementation of NS1 replication defects depends on the degree of the deletion, with at least 54 carboxy-terminal amino acid residues being required in *cis* for efficient rescue, whereas bigger deletions could not be complemented [16,19,39,53]. This observation is similar to our results and suggests that the carboxy-terminal region might play an important role in a *cis*-dominant process such as polyprotein cleavage. Alternatively, based on the recent identification of multiple host ribosomal proteins and chaperones as NS1 interaction partners [28], it is conceivable that this domain might contain binding sites for host cell factors required for stability or folding of the (immature) polyprotein, thus contributing indirectly to the formation of a functional replication complex. This hypothesis would be in agreement with the lower levels of some NS proteins observed upon expression of the DENV polyprotein with the complete NS1 deletion. Alternatively, NS1 or an NS1-2A precursor [34] might be involved in stabilizing other NS proteins. Although impaired polyprotein cleavage, resulting from complete NS1 deletion, cannot be excluded, we did not observe alterations in the ratios of polyprotein cleavage products under steady-state conditions. Consistently, it has been reported that the last 8 amino acid residues of NS1 are sufficient for NS1-2A cleavage [54]. In any case, the observation that the partial deletion in NS1 can be rescued by *trans*-complementation clearly shows that remaining parts of NS1 are also indispensable for VP biogenesis. Though underlying mechanisms remain to be clarified, observed functions of NS1 might result from an intrinsic membrane-bending ability of the central region in NS1, its affinity for lipids that are recruited to the site of the viral replicase or interactions with host proteins mediating these or some other function.

In summary, our study identifies a novel interaction between NS1 and the NS4A-2K-NS4B cleavage intermediate which plays a critical role in the DENV replication cycle. In addition, we provide evidence for a contribution of NS1 to the formation of the membranous replication organelle that is independent from viral RNA replication. Finally, we establish a comprehensive map of regions and domains involved in the various functions of NS1. The multitude of roles fulfilled by NS1, including its contribution to the pathogenesis of dengue [21], identifies NS1 as a highly promising target for direct acting antivirals aiming to suppress viral replication and severe disease manifestations [55]. Although further investigations will be needed to mechanistically define the various functions NS1 exerts in the viral replication cycle, the genetic map established in the present study offers a starting point for the design of antiviral agents targeting this DENV “Swiss Army Knife” [56].

## Materials and methods

### Cell lines

All cell lines were maintained in Dulbecco's modified Eagle medium (DMEM; ThermoFisher Scientific, Darmstadt, Germany) supplemented with 2 mM L-glutamine, nonessential amino acids, 100 U penicillin/ml, 100 μg streptomycin/ml, and 10% fetal calf serum. VeroE6 cells [57] were obtained from Progen (Heidelberg Germany). Huh7 cells [58] were obtained from the laboratory of Heinz Schaller (Center for Molecular Biology, Heidelberg). These cells served as founder for the production of all Huh7 derived subclones. Huh7_T7 and Huh7-Lunet_T7 cells [30] were generated by lentiviral transduction to allow stable expression of the bacteriophage T7 RNA polymerase. Huh7_T7_NS2B3 cells that in addition stably express full length DENV NS2B-3 were described previously [31]. Huh7_NS1_HA and Huh7_NS1_nt were generated by transduction with lentiviral vectors encoding DENV-2 NS1 with a carboxy-terminal HA-tag or non-tagged NS1, respectively [17]. Huh7-Lunet_T7_NS1-mCh cells stably expressing NS1 with a carboxy-terminal mCherry tag were generated by transduction with lentiviral vectors encoding NS1-mCherry [17]; control Huh7-Lunet_T7_ cells were generated by transduction with the empty pWPI vector. Huh7_T7 and Huh7-Lunet_T7 cells were maintained in medium containing 5 μg/ml zeocin. Huh7-T7_NS2B3, Huh7-Lunet-T7_NS1-mCh and Huh7-LunetT7_vector cells were cultured in medium containing 5 μg/ml zeocin and 1 μg/ml puromycin and Huh7_NS1_HA and Huh7_NS1_nt cells in medium containing 1 μg/ml puromycin. All cell lines are routinely tested for mycoplasma contamination using the MycoAlert mycoplasma detection kit (Lonza, Basel, Switzerland).

### Plasmids

The plasmids pFK_DVR2A containing a DENV genome based on the 16881 strain and encoding a *Renilla* luciferase (RLuc) reporter gene as well as pFK_sgDVH2A containing a hygromycin-B selectable DENV subgenomic replicon were described previously [59]. The plasmid pFK_DVR2A^pΔNS1^ containing an in-frame deletion of 97 amino acids in NS1 was described previously [17]. The DVR2A construct containing an HA-tag within NS1 (pFK_DVR2A-NS1_HA*) was based on the insertion site reported earlier [25] and generated by overlap PCR followed by insertion of the amplicon via KasI and MluI restriction sites into pFK_DVR2A. DVR2A constructs containing pseudoreversions identified in this study were created by overlap PCR and insertion of PCR products into wildtype DVR2A or DVR2A containing a specified primary NS1 mutation. All NS1 constructs used in this study contain the last 72 nucleotides of E (ET) immediately upstream of the NS1 sequence to ensure proper insertion into the ER membrane and signalase cleavage. Lentiviral expression constructs pWPI_puro_NS1_HA, pWPI_NS1_mCh and pWPI_puro_NS1_nt containing the DENV-2 (16681) NS1 sequence with or without carboxy-terminal tag were described previously [17]. Expression constructs pTM_NS1_HA (containing a carboxy-terminal HA-tag) and pTM_NS1_nt were generated by PCR using pCDNA_NS1_HA and pCDNA_NS1_nt as template, respectively. XmaI and BamHI restricted amplicons were inserted into the pTM expression vector [60]. Expression constructs pTM_NS4A-2K-4B and pTM_2K-4B were described previously [31]. Plasmid pTM_FLAG_NS4A was generated by PCR using pTM_NS4A-2K-4B as template followed by insertion of the NcoI and BamHI restricted PCR products into the pTM vector. NS1 point mutations were inserted into the construct pSM3-DVs_CAE_NS1-3’ encoding the complete DENV polyprotein under the control of the T7 RNA polymerase promoter by using the DVR2A constructs containing the desired mutations and transfer of MluI—KasI DNA fragments or by overlap PCR in the case of mutations located upstream of the MluI site. The polyprotein construct containing an internally HA-tagged NS1 (pSM3-DVs_CAE_NS1-3’_NS1_HA*) was generated by inserting the NS1-NS2A fragment isolated from pFK_DVR2A-NS1_HA* via KasI and MluI sites into pSM3-DVs_CAE_NS1-3’. The polyprotein construct containing a complete NS1 deletion (pSM3-DVs_CAE_NS1-3’_cΔNS1) was obtained by overlap PCR, generating a DNA fragment containing the last 24 codons of E and the first 5 codons of NS1 directly fused to last 8 codons of NS1 that was inserted via BamHI and KasI sites into pSM3-DVs_CAE_NS1-3’ plasmid. The polyprotein construct containing a partial deletion in NS1 (pSM3-DVs_CAE_NS1-3’_pΔNS1) was created by inserting the NS1-NS2A fragment from pFK_DVR2A^pΔNS1^ via MluI and KasI sites into pSM3-DVs_CAE_NS1-3’. The plasmid pFK_DVR2A^cΔNS1^ was generated by overlap PCR using pSM3-DVs_CAE_NS1-3’_cΔNS1 and pFK_DVR2A as templates and insertion of BamHI and KasI digested PCR products into pSM3-DVs_CAE_NS1-3’. A complete list of primers used in this study is available upon request.

### Antibodies

Rabbit antisera raised against various DENV proteins (NS1, NS2B, NS3, NS4A, NS4B and NS5) have been described previously [4] and were used at a 1:1,000 dilution. In addition, the following primary antibodies were used for immunofluorescence staining: rabbit polyclonal anti-NS4B antibody, mouse monoclonal anti-NS3 antibody, mouse monoclonal anti-NS1 antibody (all from GeneTex, Irvine, CA, USA), mouse monoclonal anti-reticulon 3 antibody (Santa Cruz, Dallas, TX, USA), and anti-protein disulfide isomerase rabbit polyclonal antibody (Cell Signaling Technology, Danvers, MA, USA). The mouse monoclonal anti-GAPDH antibody, as well as anti-rabbit and anti-mouse secondary antibodies conjugated to horseradish peroxidase were purchased from Sigma-Aldrich (Tufkirchen, Germany).

### *In vitro* transcription and viral RNA transfection

For *in vitro* transcription, 5 or 10 μg of plasmid DNA was linearized using the XbaI restriction enzyme and purified using NucleoSpin Gel and PCR Clean-up (Macherey-Nagel, Düren, Germany). *In vitro* transcription was carried out using the SP6 RNA polymerase as described previously [59]. *In vitro* transcripts were purified by phenol-chloroform extraction and resuspended in RNase-free water. RNA integrity was confirmed by agarose gel electrophoresis. For RNA transfection VeroE6 or Huh7-derived cells were trypsinized, harvested in complete DMEM and washed once with PBS. Cells were resuspended in cytomix (120 mM KCl, 0.15 mM CaCl_2_, 10 mM potassium phosphate buffer, 2 mM EGTA, 5 mM MgCl_2_, 25 mM HEPES [pH 7.6], 2 mM ATP and 5 mM glutathione, the latter two freshly added) at a density of 1.5x10^7^ Vero cells/ml or 1x10^7^ Huh7-derived cells and 400 μl of the cell suspension were mixed with 5 μg of *in vitro* transcripts. Cells were transferred into a 0.2 cm gap width electroporation cuvette (BioRad Hercules, CA, USA), pulsed once with 166 V and 500 μF, resuspended in pre-warmed complete DMEM and seeded as required for subsequent assays.

### Viral replication assays

Stocks of the DVR2A^pΔNS1^ virus containing an in-frame deletion of 97 amino acids in NS1 were produced in VeroE6 helper cells stably expressing NS1 and titrated by focus forming assay as previously described [17]. Virus stocks of DVR2A and DVR2A-NS1_HA* were produced as described previously [31] and titrated by plaque assay. Viral replication was measured in cells infected or transfected with DVR2A by using RLuc assay as described elsewhere [61]. In brief, cells grown on 24-well plates were lysed in 100 μl RLuc lysis buffer (25 mM Glycine-Glycine [pH 7.8], 15 mM MgSO_4_; 4 mM EGTA, 10% (v/v) glycerol, 0.1–1% (v/v) Triton X-100, 1 mM DTT added right before use) at indicated time points after transfection/infection, snap frozen at -70°C and thawed prior to use. RLuc activity was measured using a Mithras LB 940 plate reader (Berthold technologies, Bad Wildbad, Germany) after addition of 400 μl assay buffer (25 mM Glycine-Glycine [pH 7.8], 15 mM K_4_PO_4_ buffer [pH 7.8], 15 mM MgSO_4_, 4 mM EGTA) containing 1.43 μM of coelenterazine (PJK, Kleinblittersdorf, Germany). In some cases, RLuc activity was measured with a Lumat LB9507 tube luminometer (Berthold) after mixing 20 μl of cell lysate with 100 μl of RLuc buffer.

### Selection for pseudorevertants

Single substitutions in NS1 were inserted into a selectable subgenomic DENV replicon (sgDVH2A) containing a hygromycin phosphotransferase gene downstream of the cis-acting elements of the capsid-coding region (CS). A 2A cleavage sequence at the carboxy-terminus of the hygromycin phosphotransferase gene was inserted to allow proper processing of the DENV polyprotein. VeroE6 cells were electroporated with selectable replicon RNAs and cultured in the presence of 150–250 μg/ml Hygromycin B. After three to four weeks, single cell clones were isolated and expanded and once sufficient cell numbers had been reached, total cellular RNA was extracted using the NucleoSpin RNA II kit (Macherey-Nagel, Germany). Viral RNA was reverse transcribed using the SuperScript III reverse transcriptase (ThermoFisher Scientific) and the primer 5’-CGA CCT GAC TTC TAG CCT TGT TTC-3’. cDNA was used to amplify a DNA fragment spanning the coding region of the DENV-2 non-structural proteins (from nucleotide 2,422 to 10,248) using the Expand Long Template PCR System (Roche, Mannheim, Germany), forward primer 5’-ATT AGA GCT CGA TAG TGG TTG CGT TGT GAG CT-3’ and reverse primer 5’-ATA ATC TAG ACC ACA GAA CTC CTG CTT CTT CC-3’. Purity and integrity of amplicons was verified by agarose gel electrophoresis and excised fragments were subjected to nucleotide sequence analysis.

### Transfection of plasmid DNA

Target cells were seeded one day prior to transfection to achieve 90–100% confluency at the time point of transfection. After a medium change 30 min before transfection, cells were transfected using the Trans-IT-LT1 transfection reagent (Mirus, Madison, WI, USA), according to the protocol of the manufacturer, except for 10 cm-diameter culture dishes where 10 μg of DNA, 30 μl of Trans-IT-LT1 reagent and 800 μl transfection medium were used. Reduced serum Opti-MEM medium (ThermoFisher Scientific) was used for preparing transfection complexes. In the case of co-transfection of two constructs, equal amounts of each plasmid DNA were used to reach a total DNA amount required for the given format. In the case of EM or immunofluorescence analysis, medium was changed 4 h post transfection. Cells were lysed or fixed 16 to 20 h after transfection and processed for subsequent assays.

### Immunoprecipitation

Infected or transfected cells were washed twice with PBS, lysed in 1 to 2 ml of immunoprecipitation (IP) lysis buffer (150 mM NaCl, 50 mM Tris-HCl [pH 7.4], 0.5% Triton-X100, freshly supplemented with 1% cOmplete protease inhibitor cocktail (Roche)), and incubated on ice for 1 h. Lysates were centrifuged at 21,000 x g for 1 h. In some cases, cells were collected in PBS and dry cell pellets were stored at -80°C prior to processing for cell lysis. Pre-cleared cell lysates were normalized to the sample with lowest total protein concentration as measured by Bradford assay [62]; 10% of total normalized cell lysate was saved as input and the rest was added to 20 μl of HA-specific agarose beads slurry (Sigma-Aldrich, St. Louis, MO, USA). After incubation for 3 to 5 h at 4°C with gentle agitation, the resin was washed extensively with lysis buffer and samples were eluted once with 3% SDS in PBS and once with PBS. Eluates were collected, pooled and subjected to overnight acetone precipitation. Samples were centrifuged for 1 h at 21,000 x g, pellets were resuspended in 2 x SDS-PAGE loading buffer (230 mM Tris-HCl [pH 6,8], 120 mM SDS, 200 mM DTT, 3.5% glycerol, 0.1% bromophenol blue) and denatured by 5 min incubation at 98°C. In the case of DVR2A_NS1_HA*-infected cells the same procedure was employed but using Pierce anti-HA magnetic beads (Thermo Fisher Scientific) and direct processing of eluates without the acetone precipitation step.

### Determination of the NS1 viral interactome by quantitative LC-MS/MS proteomics

For mass spectrometry (MS) analysis, cells were processed for HA-specific affinity purification as described above. After washing with lysis buffer, proteins bound to the resin were washed additionally 3 times in lysis buffer without detergent and protease inhibitors. Bound proteins were denatured by incubation in 20 μl guanidinium chloride buffer (600 mM GdmCl, 1mM Tris[2-carboxyethyl] phosphine–HCl, 4mM chloroacetamide, 100 mM Tris-HCl [pH 8.0]). After digestion with 1 μg LysC (WAKO Chemicals USA) at room temperature for 3 h, the suspension was diluted in 100 mM Tris-HCl [pH 8.0], and the protein solution was digested with trypsin (Promega) overnight at room temperature. Peptides were purified on stage tips with three C18 Empore filter discs (3M, Maplewood, MN, USA) and analyzed by liquid chromatography coupled to MS on an Orbitrap XL instrument (Thermo Fisher Scientific) as described previously [63]. Raw MS data were processed with the MaxQuant software package, version 1.5.3 [64] using the built-in Andromeda search engine to search against the human proteome (UniprotKB, release 2012_01) containing forward and reverse sequences concatenated with the DENV polyprotein (Uniprot ID: P-29990) with the individual viral open reading frames manually annotated, and the label-free quantitation algorithm as described previously [65]. Additionally, the intensity-based absolute quantification (iBAQ) algorithm and Match Between Runs option were used. In MaxQuant, carbamidomethylation was set as fixed and methionine oxidation and N-acetylation as variable modifications, using an initial mass tolerance of 6 ppm for the precursor ion and 0.5 Da for the fragment ions. Search results were filtered with a false discovery rate (FDR) of 0.01 for peptide and protein identifications. The Perseus software package, version 1.5.3.0 was used to further process the data. Protein tables were filtered to eliminate the identifications from the reverse database and common contaminants. In analyzing MS data, only proteins identified on the basis of at least one peptide and a minimum of three quantitation events in at least one experimental group were considered. IBAQ protein intensity values were normalized against the median intensity of each sample (using only peptides with recorded intensity values across all samples and biological replicas), log-transformed and missing values filled by imputation with random numbers drawn from a normal distribution calculated for each sample. Significant interactors were determined by multiple equal variance t-tests with permutation-based false discovery rate statistics. We performed 250 permutations and the FDR threshold was set at 0.001. The parameter S0 was set at 2 to separate background from specifically enriched interactors. Results were plotted as Volcano plot and heat map using the Perseus software package [64].

### Western blots

Cells were lysed in IP lysis buffer, incubated on ice for 1 h and samples cleared by centrifugation at 21,000 x g for 1 h. Protein concentration was measured by Bradford assay, samples were denatured by boiling for 5 min in 98°C in SDS-PAGE buffer (120 mM Tris-HCl [pH 6.8], 60 mM SDS, 100 mM DTT, 1.75% glycerol, 0.1% bromophenol blue) and 20 to 30 μg of total protein was loaded onto each lane of a gel. Proteins were separated by SDS-PAGE and transferred onto a polyvinylidenfluorid membrane. After blocking of the membrane with 5% milk in PBS-T (PBS with 0.5% Tween) or 5% BSA in PBS-T, they were incubated with primary and secondary horse radish peroxidase-conjugated antibodies as specified in the antibody section. Signals were developed by using the Western Lightning Plus-ECL reagent (Perkin Elmer, Waltham, MA, USA) and visualized with a ChemoCam Imager 3.2 (Intas Science Imaging Instruments GmbH, Göttingen, Germany). The LabImage 1D software (Intas) was used for quantification of protein-specific signals.

### Ultrastructural analysis by transmission electron microscopy

Cells grown on glass coverslips were fixed with 2% glutaraldehyde in 50 mM cacodylate buffer [pH 7,2] containing 10 mM MgCl_2_, 10 mM CaCl_2_, 100 mM KCl and 2% sucrose. Cells were either stored at 4°C for up to several days or directly washed 5x with 50 mM cacodylate buffer, incubated with 2% osmium tetroxide for 40 min on ice and 0.5% uranyl acetate, dissolved in double distilled water, for 30 min at room temperature or 24 h at 4°C. Samples were washed with double distilled water for 30 min and dehydrated step-wise with 40% to 100% ethanol, embedded in an araldite-Epon mixture (Araldite 502/Embed 812 kit; Electron Microscopy Sciences) and left for one to three days at 60°C to allow complete polymerization. After removal of the coverslip, embedded cells were cut into 70-nm thick sections with a Leica Ultracut UCT microtome (Leica, Wetzlar, Germany) and a diamond knife and mounted onto a mesh grid. Retrieved sections were further incubated with 3% uranyl acetate in 70% methanol for 5 min, followed by 2% lead citrate in distilled water for 2 min. Samples were analyzed with an EM10 transmission electron microscope (Carl Zeiss AG, Oberkochen, Germany) or a Jeol JEM-1400 (Jeol Ltd., Tokyo, Japan). For quantification of VPs, cells from randomly selected areas of the grid were analyzed. Wildtype samples were always prepared in parallel and only experiments where at least 20% of the wildtype cells were positive for VPs were taken under consideration. For each experiment the number of counted cells is given in the figure legend.

### Immunofluorescence

For immunofluorescence analysis cells grown and transfected as described above were fixed in 4% paraformaldehyde for 15 min and permeabilized by 15 min incubation with 0.2% Triton X-100 in PBS. Cells were stained with primary antibodies as specified in the figure legends, followed by staining with anti-mouse or anti-rabbit secondary antibodies, conjugated with Alexa Fluor 488 or 568 (ThermoFisher Scientific). Coverslips were mounted on glass slides and analyzed with a Nikon Eclipse Ti microscope (Nikon, Tokyo, Japan) to assess transfection efficiency, or a Leica SP8 confocal microscope (Leica) to analyze subcellular localization of DENV proteins.

### Statistical analysis and molecular graphics

Statistical analyses were performed using the GraphPad Prism 5.0 software package (LaJolla, CA, USA). Two-tailed paired Student’s t-test with Bonferroni correction for multiple samples comparison was used to assess statistical significance. All molecular graphics were prepared with either MOE 2015 or UCSF Chimera software [66].

## Supporting information

S1 Fig Replication fitness of the DENV genome encoding NS1 with an internal HA tag. Huh7 cells were electroporated with *in vitro* transcribed RNAs of full length DVR2A containing internally HA-tagged NS1 (NS1_HA*). (A) Cells were lysed at indicated time points after transfection and RLuc activity was measured to determine viral replication. (B) Supernatants from electroporated cells were harvested at indicated time points after transfection and used to infect naïve Huh7 cells. Cells were harvested 48 h post infection and RLuc activity levels were determined in cell lysates. The DVR2A genome with the complete NS1 deletion (DVR2A-cΔNS1) was used to determine the background of the assay.
(TIF)

S2 Fig Analysis of HA-NS1 immune purified protein complexes isolated from transfected cells by using a commercial NS4B-specifc antibody. Huh7-derived cells stably expressing the T7 RNA polymerase and DENV NS2B-3 were co-transfected with NS1_HA or non-tagged NS1 (NS1_nt) and processed for immunoprecipitation as described in Fig 4. Captured protein complexes were analyzed by immunoblotting using a commercial NS4B antiserum (GeneTex) and the NS1-specific rabbit antiserum.
(TIF)

S3 Fig Evidence that the NS4A-2K-4B precursor is not glycosylated. Given the recent report of NS4B glycosylation, potential glycosylation of the NS4A-2K-4B cleavage intermediate was assessed by performing PNGase-F treatment. Huh7-derived cells stably expressing the T7 RNA polymerase and DENV NS2B-NS3 were co-transfected with plasmids encoding HA-tagged or non-tagged NS1 and NS4A-2K-4B. Cells were harvested 16 h post transfection and lysates used for HA-specific pull-down. Eluates were concentrated by acetone precipitation. Protein complexes were dissolved in water and treated with PNGase F (NEB, Ipswich, MA, USA) under denaturing conditions according to the manufacturer’s protocol. Mock-treated samples were prepared in parallel. Samples were analyzed by western blot using antibodies specified on the right. While PNGase treatment of NS1 increased its electrophoretic mobility, indicating removal of glycosylation, we could not observe a shift in case of the NS4B-containing proteins, suggesting that they are not glycosylated. Note that in the samples processed for deglycosylation, the ~35 kDa precursor band (see Fig 4) was no longer visible, neither in PNGase-treated nor control samples. Since the PNGase deglycosylation protocol requires boiling of the samples at 100°C for 10 min, we assume that this treatment results in full denaturation of the ~35 kDa protein species which then migrates like the ~30 kDa protein species. The protein with an apparent molecular weight of ~60 kDa might represent a heat stable dimer of NS4A-2K-4B.
(TIF)

S4 Fig Effect of second-site mutations in NS4B on NS1 –NS4A-2K-NS4B interaction. Huh7 cells stably expressing the T7 RNA polymerase and proteolytically active DENV NS2B-3 were co-transfected with constructs encoding HA-tagged wildtype (WT) or mutated NS1 and the wildtype or mutated NS4A-2K-4B polyprotein construct. Cell lysates were processed as described in Fig 4B and immunoblots were probed with NS1- and NS4B-specific antisera.
(TIF)

S5 Fig Subcellular localization of NS3 and NS4B in cells transfected with the DENV-2 polyprotein expression constructs containing deletions in NS1. Huh7-Lunet_T7 cells were grown on coverslips, transfected with constructs encoding the wildtype (WT), pΔNS1, or cΔNS1 polyprotein and fixed 18 h post transfection. Proteins were detected using antibodies against NS3 or NS4B and PDI (protein disulfide isomerase) or RTN3 (reticulon 3), which both are ER markers, respectively. Note that two different NS4B staining patterns, i.e. diffuse or punctuated, were observed in both WT and ΔNS1 transfected cells. Scale bar = 20 μm.
(TIF)

S6 Fig Homology model of the NS1 dimer. The model is based on the DENV NS1 structure (PDB 4O6B) with missing residues modelled based on the ZIKV NS1 structure (PDB 5K6K) as described in Fig 1(A). Amino acid residues involved in interaction with the NS4A-2K-4B precursor are marked in blue. Residues required for replication and residing close to the proposed membrane interface, but having no impact on NS1 interaction with NS4A-2K-4B are marked in red.
(TIF)
