# Supplementary figures and images for: A novel interaction between dengue virus nonstructural protein 1 and the NS4A-2K-4B precursor is required for viral RNA replication but not for formation of the membranous replication organelle
Source: PLoS Pathog. 2019 May 9;15(5):e1007736. doi: 10.1371/journal.ppat.1007736 (PMC6508626; doi:10.1371/journal.ppat.1007736)

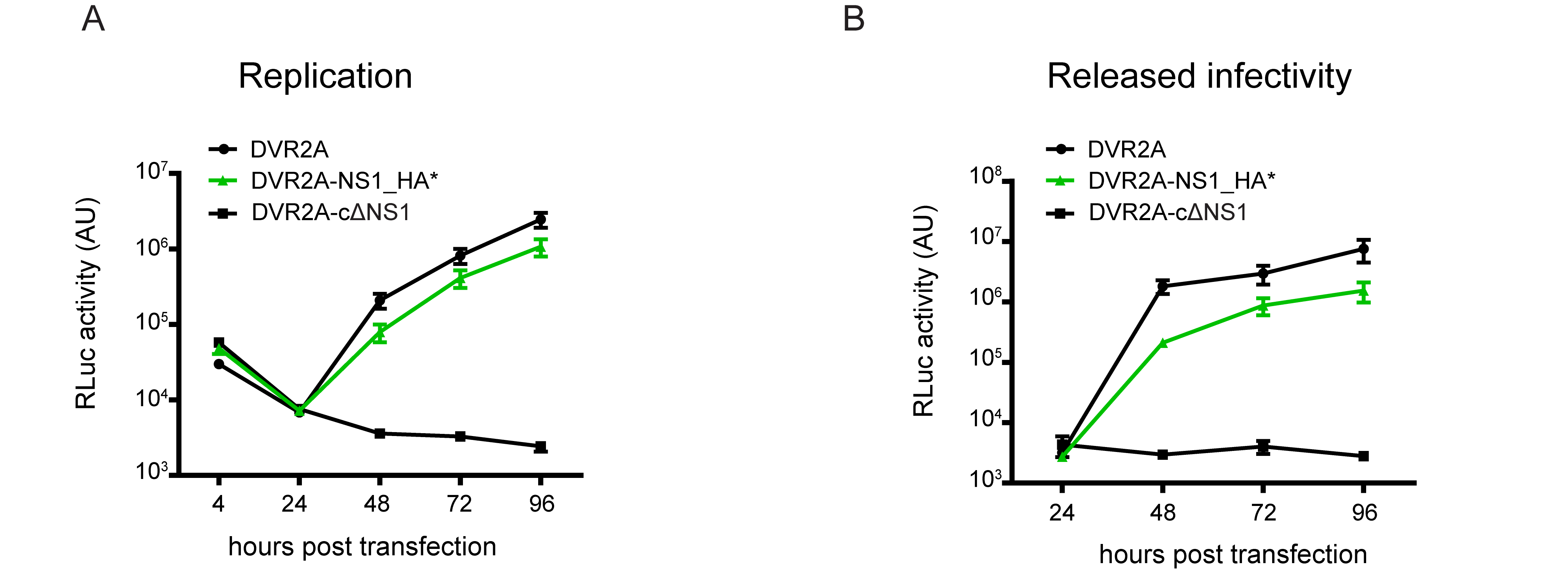

Supplement: S1 Fig — Huh7 cells were electroporated with in vitro transcribed RNAs of full length DVR2A containing internally HA-tagged NS1 (NS1_HA*). (A) Cells were lysed at indicated time points after transfection and RLuc activity was measured to determine viral replication. (B) Supernatants from electroporated cells were harvested at indicated time points after transfection and used to infect naïve Huh7 cells. Cells were harvested 48 h post infection and RLuc activity levels were determined in cell lysates. The DVR2A genome with the complete NS1 deletion (DVR2A-cΔNS1) was used to determine the background of the assay. (TIF) [file ppat.1007736.s001.tif]

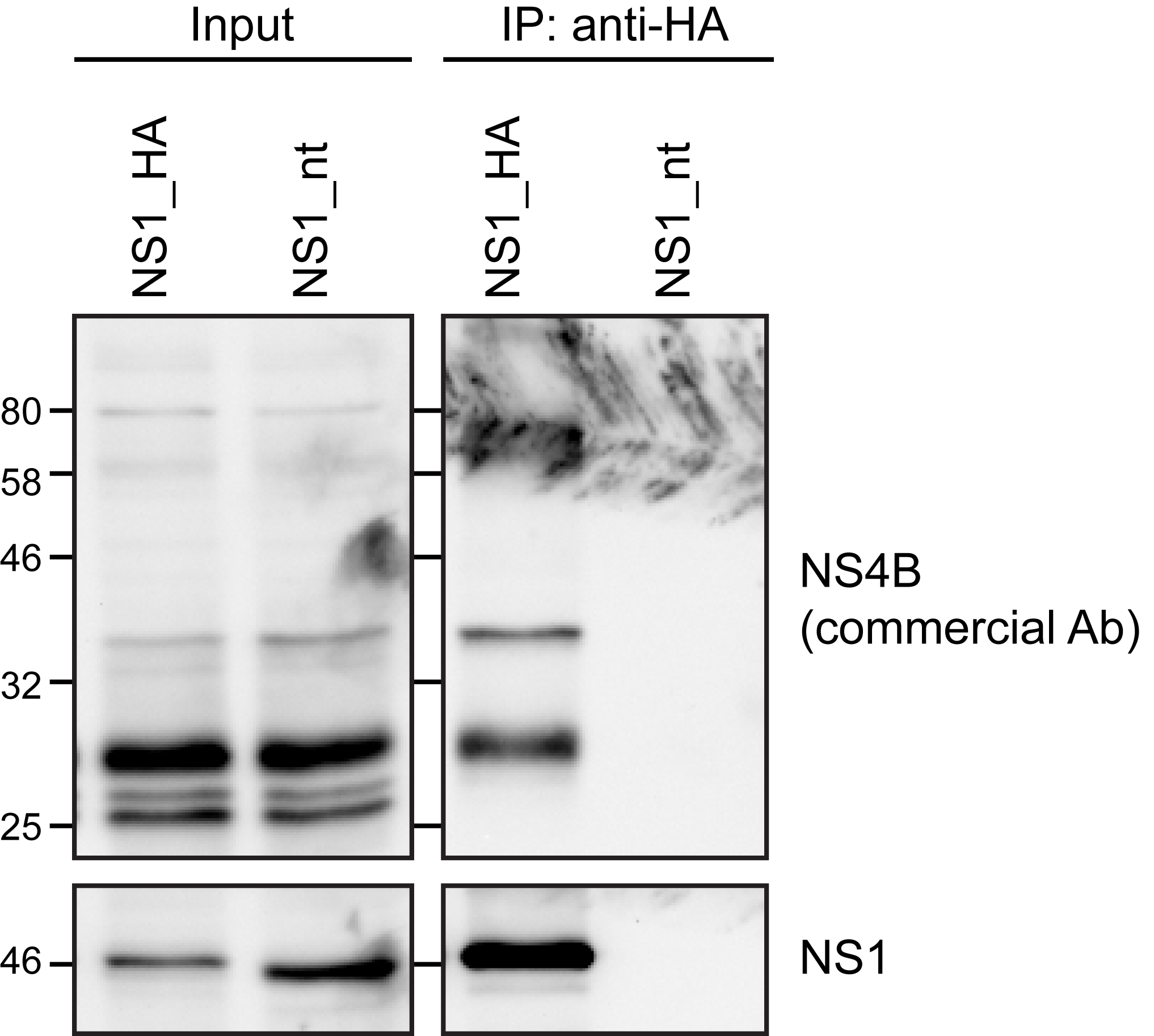

Supplement: S2 Fig — Huh7-derived cells stably expressing the T7 RNA polymerase and DENV NS2B-3 were co-transfected with NS1_HA or non-tagged NS1 (NS1_nt) and processed for immunoprecipitation as described in Fig 4. Captured protein complexes were analyzed by immunoblotting using a commercial NS4B antiserum (GeneTex) and the NS1-specific rabbit antiserum. (TIF) [file ppat.1007736.s002.tif]

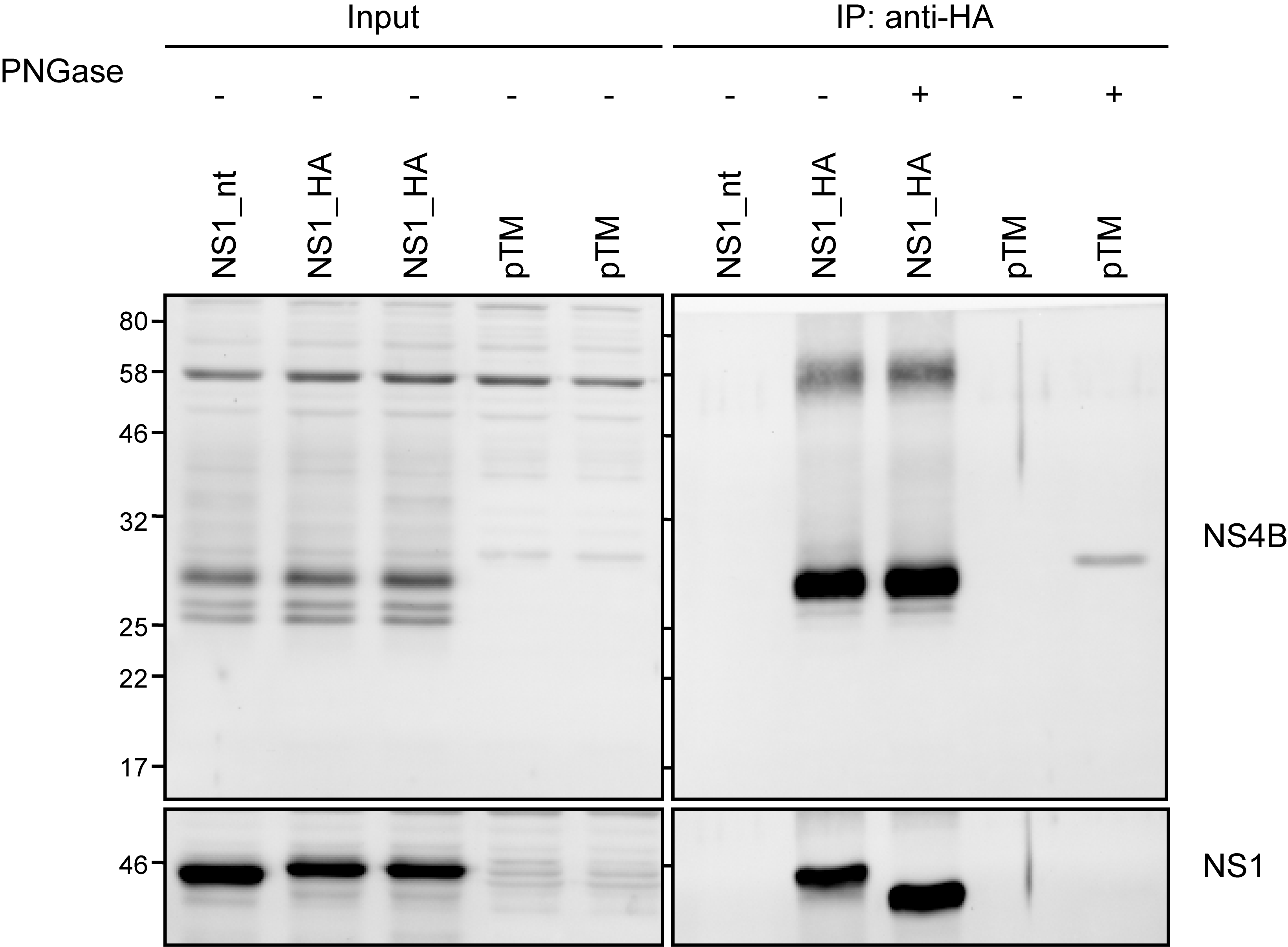

Supplement: S3 Fig — Given the recent report of NS4B glycosylation, potential glycosylation of the NS4A-2K-4B cleavage intermediate was assessed by performing PNGase-F treatment. Huh7-derived cells stably expressing the T7 RNA polymerase and DENV NS2B-NS3 were co-transfected with plasmids encoding HA-tagged or non-tagged NS1 and NS4A-2K-4B. Cells were harvested 16 h post transfection and lysates used for HA-specific pull-down. Eluates were concentrated by acetone precipitation. Protein complexes were dissolved in water and treated with PNGase F (NEB, Ipswich, MA, USA) under denaturing conditions according to the manufacturer’s protocol. Mock-treated samples were prepared in parallel. Samples were analyzed by western blot using antibodies specified on the right. While PNGase treatment of NS1 increased its electrophoretic mobility, indicating removal of glycosylation, we could not observe a shift in case of the NS4B-containing proteins, suggesting that they are not glycosylated. Note that in the samples processed for deglycosylation, the ~35 kDa precursor band (see Fig 4) was no longer visible, neither in PNGase-treated nor control samples. Since the PNGase deglycosylation protocol requires boiling of the samples at 100°C for 10 min, we assume that this treatment results in full denaturation of the ~35 kDa protein species which then migrates like the ~30 kDa protein species. The protein with an apparent molecular weight of ~60 kDa might represent a heat stable dimer of NS4A-2K-4B. (TIF) [file ppat.1007736.s003.tif]

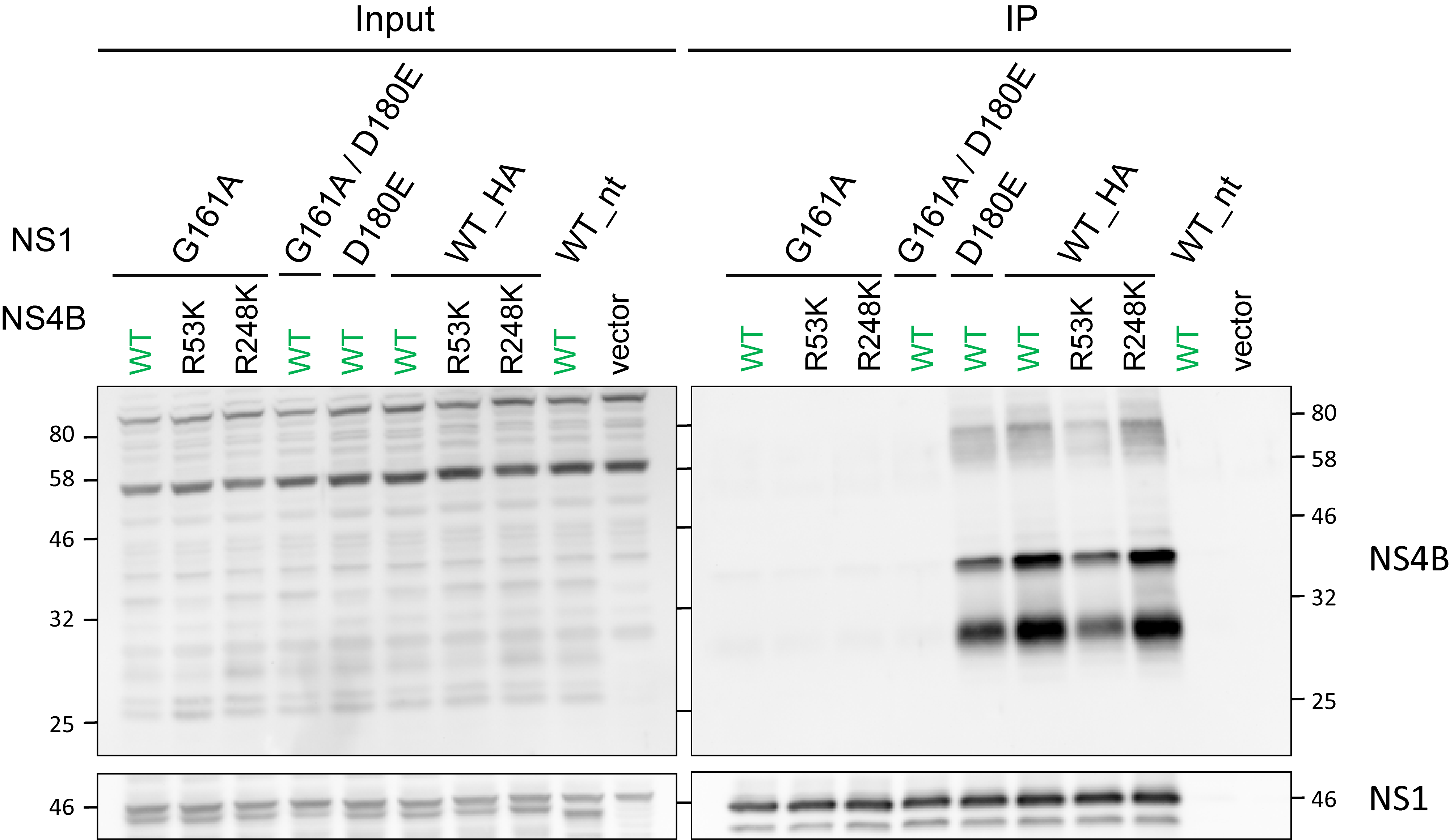

Supplement: S4 Fig — Huh7 cells stably expressing the T7 RNA polymerase and proteolytically active DENV NS2B-3 were co-transfected with constructs encoding HA-tagged wildtype (WT) or mutated NS1 and the wildtype or mutated NS4A-2K-4B polyprotein construct. Cell lysates were processed as described in Fig 4B and immunoblots were probed with NS1- and NS4B-specific antisera. (TIF) [file ppat.1007736.s004.tif]

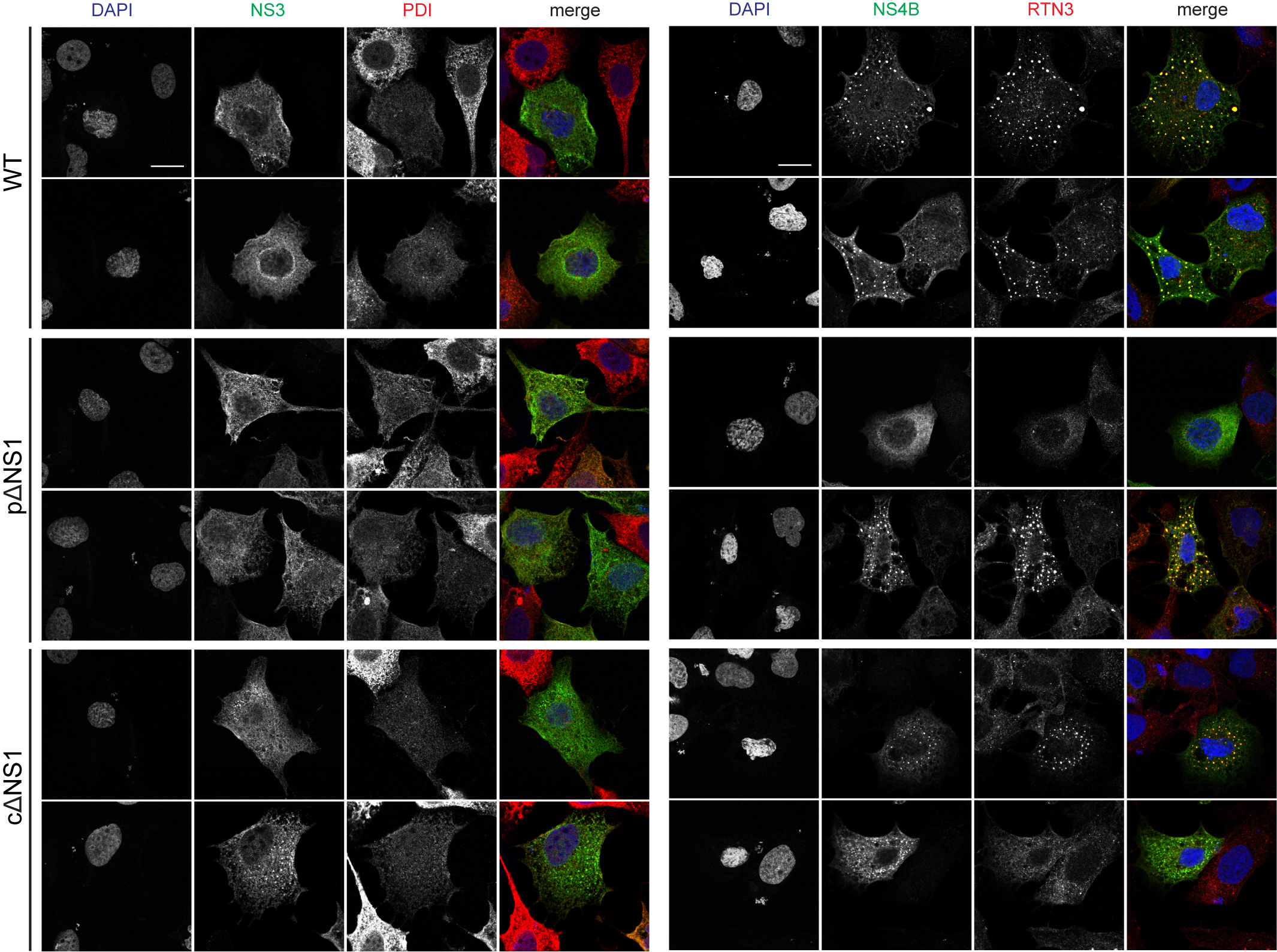

Supplement: S5 Fig — Huh7-Lunet_T7 cells were grown on coverslips, transfected with constructs encoding the wildtype (WT), pΔNS1, or cΔNS1 polyprotein and fixed 18 h post transfection. Proteins were detected using antibodies against NS3 or NS4B and PDI (protein disulfide isomerase) or RTN3 (reticulon 3), which both are ER markers, respectively. Note that two different NS4B staining patterns, i.e. diffuse or punctuated, were observed in both WT and ΔNS1 transfected cells. Scale bar = 20 μm. (TIF) [file ppat.1007736.s005.tif]

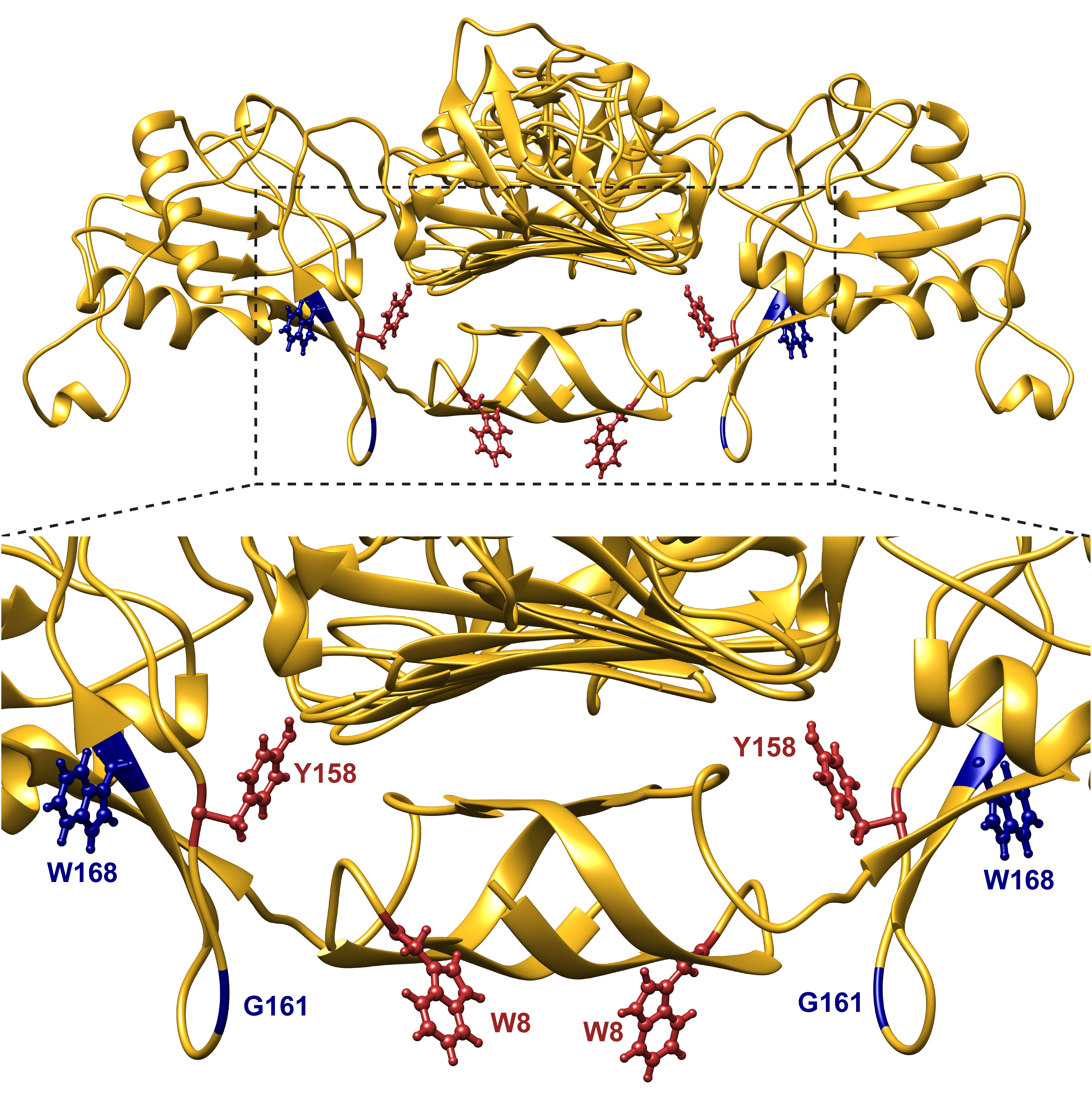

Supplement: S6 Fig — The model is based on the DENV NS1 structure (PDB 4O6B) with missing residues modelled based on the ZIKV NS1 structure (PDB 5K6K) as described in Fig 1(A). Amino acid residues involved in interaction with the NS4A-2K-4B precursor are marked in blue. Residues required for replication and residing close to the proposed membrane interface, but having no impact on NS1 interaction with NS4A-2K-4B are marked in red. (TIF) [file ppat.1007736.s006.tif]
